# Nitroxyl Radical as a Theranostic Contrast Agent in Magnetic Resonance Redox Imaging

**DOI:** 10.1089/ars.2021.0110

**Published:** 2022-01-17

**Authors:** Ken-ichiro Matsumoto, Ikuo Nakanishi, Zhivko Zhelev, Rumiana Bakalova, Ichio Aoki

**Affiliations:** ^1^ Quantitative RedOx Sensing Group, Department of Radiation Regulatory Science Research, National Institute of Radiological Sciences, Quantum Life and Medical Science Directorate, National Institutes for Quantum and Radiological Science and Technology, Chiba-shi, Japan.; ^2^ Quantum RedOx Chemistry Group, Institute for Quantum Life Science, Quantum Life and Medical Science Directorate, National Institutes for Quantum and Radiological Science and Technology, Chiba-shi, Japan.; ^3^ Medical Faculty, Trakia University, Stara Zagora, Bulgaria.; ^4^ Institute of Biophysics and Biomedical Engineering, Bulgarian Academy of Sciences, Sofia, Bulgaria.; ^5^ Functional and Molecular Imaging Goup, Department of Molecular Imaging and Theranostics, Institute for Quantum Medical Science, Quantum Life and Medical Science Directorate, National Institutes for Quantum and Radiological Science and Technology, Chiba-shi, Japan.

**Keywords:** theranostics, redox imaging, nitroxyl radical, redox-sensitive contrast agent, magnetic resonance imaging, electron paramagnetic resonance

## Abstract

***Significance:*** *In vivo* assessment of paramagnetic and diamagnetic conversions of nitroxyl radicals based on cyclic redox mechanism can be an index of tissue redox status. The redox mechanism of nitroxyl radicals, which enables their use as a normal tissue-selective radioprotector, is seen as being attractive on planning radiation therapy.

***Recent Advances:*** *In vivo* redox imaging using nitroxyl radicals as redox-sensitive contrast agents has been developed to assess tissue redox status. Chemical and biological behaviors depending on chemical structures of nitroxyl radical compounds have been understood in detail. Polymer types of nitroxyl radical contrast agents and/or nitroxyl radical-labeled drugs were designed for approaching theranostics.

***Critical Issues:*** Nitroxyl radicals as magnetic resonance imaging (MRI) contrast agents have several advantages compared with those used in electron paramagnetic resonance (EPR) imaging, while support by EPR spectroscopy is important to understand information from MRI. Redox-sensitive paramagnetic contrast agents having a medicinal benefit, that is, nitroxyl-labeled drug, have been developed and proposed.

***Future Directions:*** A development of suitable nitroxyl contrast agent for translational theranostic applications with high reaction specificity and low normal tissue toxicity is under progress. Nitroxyl radicals as redox-sensitive magnetic resonance contrast agents can be a useful tool to detect an abnormal tissue redox status such as disordered oxidative stress. *Antioxid. Redox Signal*. 36, 95–121.

## Introduction

The term “redox” is a word created by combining reduction and oxidation. Reduction means receiving an electron and oxidation means losing an electron; therefore, reduction and oxidation, that is, a redox reaction, occur simultaneously. Redox is the exchange of an electron between molecules. All life is maintained by systematically regulated redox reactions. An exogenous compound introduced into a living cell system is reduced or oxidized according to the tissue redox status/balance. The tissue redox status is an index of activity or condition of cells constituting the tissue. A hypoxic environment in cancer/tumor tissue produced by uncontrolled vasculature may shift the redox balance in the tissues/cells (18, 36, 134). Collapsed vasculatures in wound tissue make tissue hypoxic and shift the redox status in tissue (82, 141, 160). Pathological conditions in tissues/cells, such as inflammation, can also alter the tissue redox status (63, 130).

Redox imaging, which can visualize the redox status of a target tissue/organ noninvasively, was developed as a diagnostic technique for analyzing redox physiology, investigating oxidative stress in animal pathophysiological models, and improving radiation therapy. Redox sensing using nitroxyl radicals was initially developed in the field of *in vivo* electron paramagnetic resonance (EPR) spectroscopy (3, 46, 65, 132). The historical development of *in vivo* EPR instruments and techniques was well summarized in previous review articles (10, 169). Nitroxyl radicals are stable free radical species when dry or dissolved in pure water, and they are directly detectable by EPR at room temperature. The nitroxyl radicals are reduced to corresponding hydroxylamines when administered to a living experimental animal (64, 121, 131). The rate constant of this *in vivo* reduction of nitroxyl radicals varies according to oxidative stress (144, 178, 184). An unbalanced tissue redox status, that is, faster or slower reduction rate of nitroxyl reduction beyond a normal range, can be considered an index of failure of redox metabolism in the tissue (102).

Since the early 2000s, nitroxyl radicals have been used as a redox-sensitive contrast agent, which was so-called spin probe at that time, in EPR imaging (EPRI) experiments (99, 104, 180, 189). When combined with EPRI, the distribution and time course of nitroxyl contrast agents in tissues can be observed (118, 123, 166). Although the temporal resolution of EPRI in that time was in the order of minutes due to the magnetic field scans based on continuous wave (CW) modality, the current temporal resolution of CW EPRI has markedly improved (159).

Nitroxyl radical compounds, which have an unpaired electron, are paramagnetic species and exert T_1_-shortening effects on nuclear spin similar to gadolinium compounds. Therefore, nitroxyl radical in an aqueous sample can be detected by enhanced T_1_-weighted contrast on magnetic resonance imaging (MRI). Mapping of the *in vivo* redox status of a target tissue, such as cancer/tumor, can be achieved by T_1_-weighted MRI with nitroxyl radicals as the contrast agent (119). Spatial and temporal high-resolution redox mapping of a particular slice is possible with excellent anatomical information from MRI.

Overhauser-enhanced MRI (OMRI) or proton electron double resonance imaging (PEDRI) is another magnetic resonance technique for imaging nitroxyl radicals *in vivo* (123, 166). These techniques can produce and observe dynamic nuclear polarization (DNP) effect *in vivo* by injecting a nitroxyl radical compound into experimental animals (24, 41, 186). Nitroxyl compounds are not suitable for producing better DNP effects due to the relatively broad EPR line width, that is, fast relaxation time, even using a ^15^N-labeled nitroxyl radical compound. However, OMRI can observe spectral information based on EPR and high image resolution based on MRI, and therefore this multimodality is advantageous for future theranostic approaches.

In this review, the authors describe the detection and analysis of redox reactions using a stable nitroxyl radical probe *in vitro* and *in vivo*. Recent applications of imaging the tissue redox status using stable nitroxyl radicals as redox-sensitive MRI contrast agents are also discussed. In addition, theranostic approaches and/or translational applications of magnetic resonance (MR) redox imaging techniques are introduced.

## Chemistry and Redox Properties of Nitroxyl Radicals

**FIG. 1.**
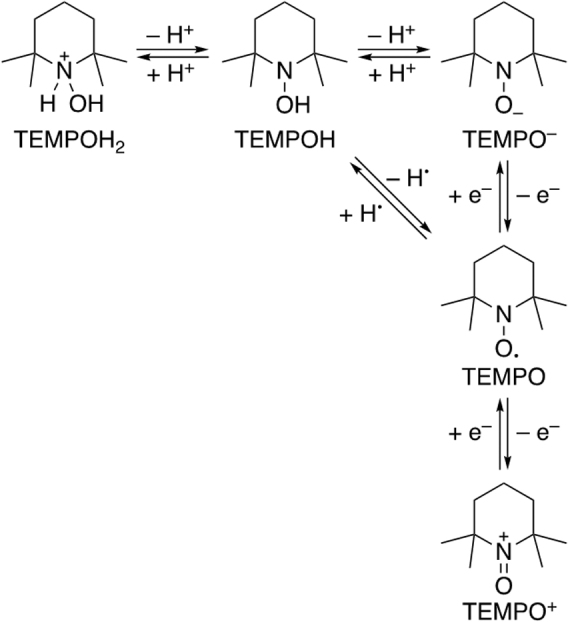
**Redox and acid–base chemistry of TEMPO.** TEMPO, 2,2,6,6-tetramethylpiperidine-*N*-oxyl.

Cyclic nitroxyl radicals with methyl groups at the carbons adjacent to the nitrogen are stable under ambient conditions and exhibit unique redox properties. Recently, their detailed chemical and electrochemical properties were reviewed (105, 106, 142). They (2,2,6,6-tetramethylpiperidine-*N*-oxyl: TEMPO as a representative example) undergo reversible one-electron oxidation reactions to produce the corresponding oxoammonium cations (oxoammonium form of TEMPO [TEMPO^+^]) (Fig. 1). A reversible cyclic voltammogram was first reported for TEMPO in 1973 (177). The six-membered ring nitroxyl radicals are generally oxidized at lower potentials than the five-membered ring compounds (11, 76, 111). The greater flexibility of the six-membered ring enables the nitrogen center to planarize more easily upon oxidation than the five-membered ring. The electron-donating groups on the ring stabilize the positive charge on the oxoammonium cations, resulting in the negative shift in the oxidation potentials. Furthermore, a significant negative shift in the oxidation potentials of carbamoyl-PROXYL (3-carbamoyl-2,2,5,5-tetramethylpyrrolidine-*N*-oxyl) and TEMPOL (4-hydroxy-2,2,6,6-tetramethylpiperidine-*N*-oxyl or 4-hydroxy-TEMPO) was reported with increasing solvent polarity (111). The stabilization of the oxoammonium cations by the solvent *via* dipolar interaction leads to a negative shift. The effects of the structure of the nitroxyl radicals on the oxidation potentials have been examined, and a good correlation was demonstrated between experimental oxidation potentials and theoretical values estimated by density functional theory calculations (108, 193).

Cyclic nitroxyl radicals were reported to undergo chemical oxidation by hydroxyl radicals (^•^OH), hydroperoxyl radical (HO_2_^•^), RO_2_^•^, ^•^NO_2_, and CO_3_^•−^ (55, 56, 58, 155). The rate constants correlate with their oxidation potentials. The involvement of electron transfer in the reaction of carbamoyl-PROXYL with cumylperoxyl radical was demonstrated by the acceleration in the presence of a redox-inactive metal ion (137), which is known to act as a catalyst in the electron transfer reactions (48, 49).

**FIG. 2.**
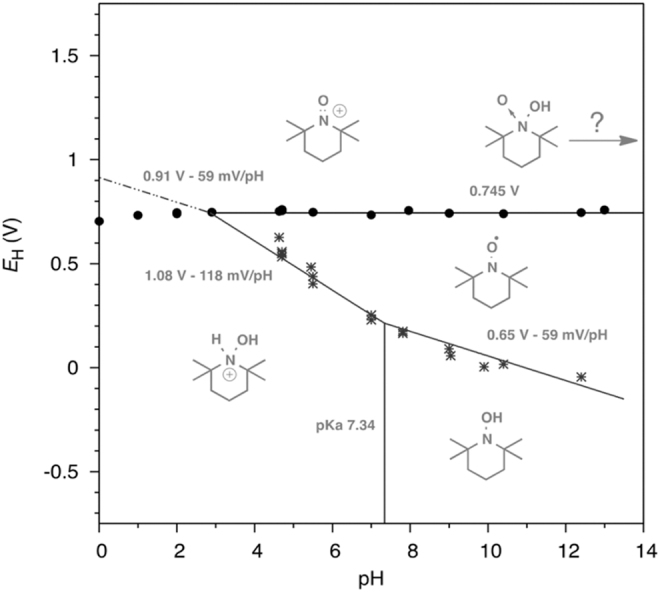
**Pourbaix diagram of TEMPO in buffered aqueous solutions.** [Adapted with permission from Gerken *et al*. (52). Copyright 2018 American Chemical Society.]

The reduction processes of the nitroxyl radicals strongly depend on the pH of the solution (89) because they are coupled with proton transfer (Fig. 1). The electrochemical reduction of the cyclic nitroxyl radicals has also been reviewed in detail (105, 106, 142). A marked change in the cyclic voltammograms depending on pH was reported for the reduction of several cyclic nitroxyl radicals (89). Recently, Stahl and colleagues reported a full Pourbaix diagram of TEMPO (Fig. 2), providing valuable information about the pH dependence of its redox properties (52). The reduction potentials of TEMPO shift to the negative direction as the pH of the solution increases, whereas the oxidation potential is pH insensitive.

The 6-membered ring nitroxyl radicals are also reduced at lower potentials than the 5-membered ring radicals due to the flexibility of the ring structure. Furthermore, the substituent effects on the reduction potentials of cyclic nitroxyl radicals were reported (112, 133).

The cyclic nitroxyl radicals are chemically reduced to the corresponding hydroxylamines by biological reductants such as ascorbate (12, 53). Yamada and colleagues demonstrated that the reduction rates of cyclic nitroxyl radicals by ascorbate correlate well with their reduction potentials and Gibbs free energy changes (Δ*G*) (185). When the Δ*G* is negative, reduction of the nitroxyl radicals by ascorbate occurs spontaneously, whereas no reduction occurs in the case of a positive Δ*G*. The involvement of hydrogen tunneling at room temperature was reported based on large kinetic isotope effects (KIE, *k*_H_/*k*_D_) in the reduction of TEMPO by ascorbate in water (*k*_H_/*k*_D_ = 24.2) and water–dioxane mixed solvent (1:1 v/v; *k*_H_/*k*_D_ = 31.0) at room temperature (87, 153). A large KIE of 12.8 was also observed for the reaction of ascorbate with 2-phenyl-4,4,5,5-tetramethylimiazolione-1-oxide (PTIO^•^) in a phosphate buffer solution at ambient temperatures (138). Thus, quantum tunneling plays a role in the reduction of nitroxyl radicals by ascorbate.

**FIG. 3.**
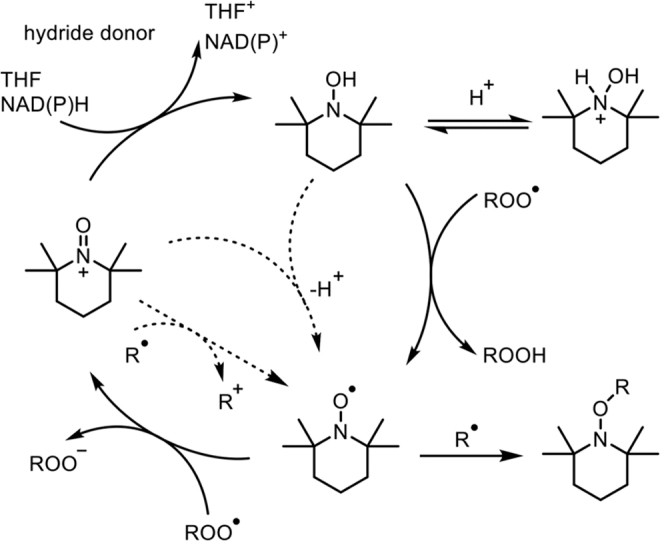
**Redox cycle of TEMPO in the reaction of ROO^•^.** ROO^•^, peroxyl radical. [Adapted with permission from Griesser *et al*. (62). Copyright 2018 American Chemical Society.]

Recently, the catalytic (redox) cycle of TEMPO in the reaction with peroxyl radicals (ROO^•^) in aqueous solution was proposed by Pratt and colleagues (Fig. 3) (62). TEMPO scavenges ROO^•^ *via* electron transfer to produce TEMPO^+^ and ROO^−^. Then, a hydride donor, such as NAD(P)H or tetrahydrofuran (THF), converts TEMPO^+^ to the corresponding hydroxylamine (TEMPO-H). The conversion of TEMPO^+^ to TEMPO-H by NADH was demonstrated to be *via* a two-electron transfer reaction (55). TEMPO-H can also scavenge ROO^•^ *via* hydrogen-atom transfer to regenerate TEMPO. However, turnover is limited at a low pH due to the protonation of TEMPO-H because the p*K*_a_ of TEMPO-H_2_^+^, which is not a hydrogen-atom donor, is 7.34 (Fig. 3). Under hypoxic conditions, the reaction of R^•^ with oxygen (O_2_) to produce ROO^•^ hardly occurs, thus TEMPO is consumed faster by the reaction with R^•^ to produce TEMPO-R than under aerobic conditions. Furthermore, in the absence of O_2_, the oxidation of R^•^ by TEMPO^+^ takes place to produce R^+^ and TEMPO (bottom-left dashed arrows in Fig. 3). Under acidic conditions, a comproportionation between TEMPO^+^ and TEMPO-H occurs to produce two molecules of TEMPO (middle dashed arrow in Fig. 3).

Cyclic nitroxyl radicals were demonstrated to be irreversibly consumed in the presence of thiols (57). The oxidation of thiols by reactive radicals, such as ^•^OH, ^•^NO_2_, and CO_3_^•−^, leads to the corresponding thiyl radicals (RS^•^). RS^•^ reacts with the nitroxyl radicals (>NO^•^) to produce the adduct >NOSR. Under physiological conditions >NOSR is decomposed to the corresponding amine (>NH) (57). These redox conversions are highly informative regarding the chemical reactivity of nitroxyl radicals as *in vivo* spin probes.

## Development of Redox Sensing into Redox Imaging

### Nitroxyl radicals as a T_1_ contrast agent

In the early 1980s, the use of nitroxyl free radicals as T_1_ contrast agents in MRI was examined (13). However, nitroxyl radicals were not considered optimal as MR contrast agents due to their low T_1_-relaxivity (r_1_) and rapid *in vivo* reduction at that time. Current MRI scanners operating with a higher magnetic field, a better signal-to-noise ratio, and efficient pulse sequences make it possible to reconsider nitroxyl radicals as potential T_1_ contrast agents.

**FIG. 4.**
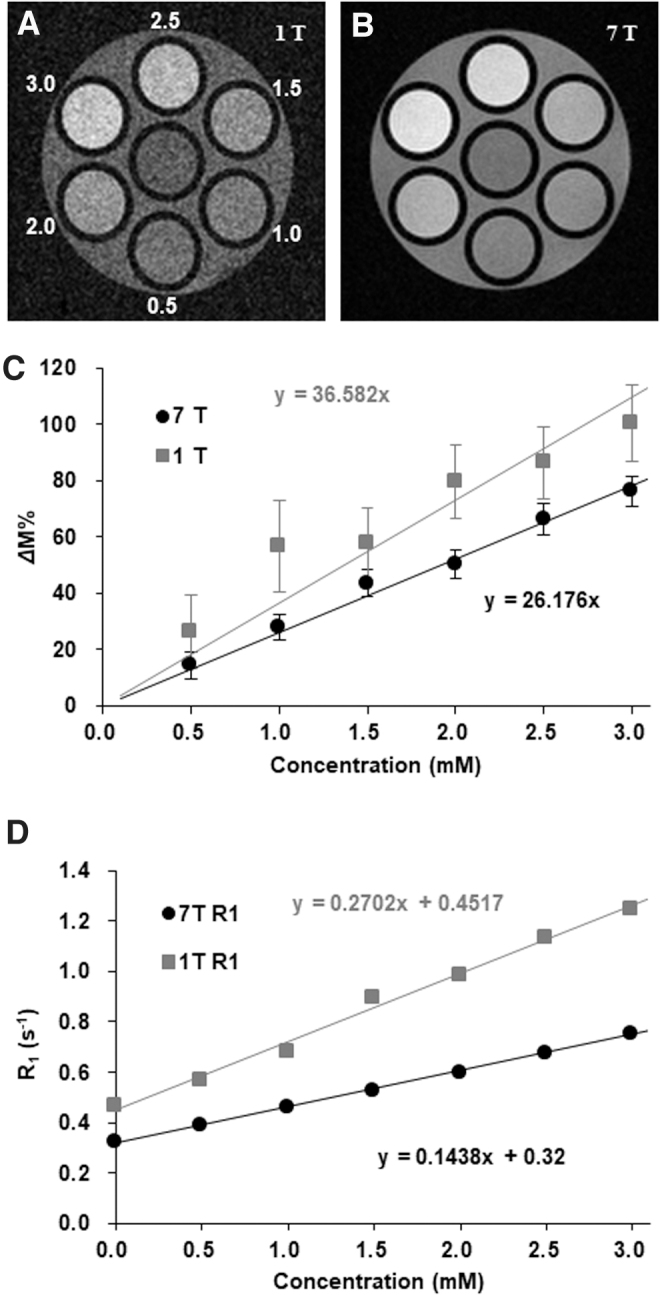
**A comparison of percentage signal amplification of T_1_-weighted image (*Δ*M%) and T_1_-relaxivity (r_1_) of a nitroxyl radical, MC-PROXYL, between different magnetic fields 1 and 7 T.** An identical 7-tube phantom was scanned by FLASH (TE = 5.1 ms, TR = 75 ms, FA = 45°), and T_1_-weighted images at **(A)** 1 T and **(B)** 7 T were obtained. The *number* indicated in **(A)** is the concentration of MC-PROXYL. The *center* tube contains water. **(C)** Relationships of *Δ*M% and concentrations of MC-PROXYL were almost linear. **(D)** T_1_ relaxation rates (R_1_) observed from T_1_ mapping of the phantom were plotted *versus* the corresponding concentration of MC-PROXYL, and r_1_ was obtained from slopes of the plots as 0.27 m*M*^−1^·s^−1^ for 1 T and 0.14 m*M*^−1^·s^−1^ for 7 T. FA, flip angle; FLASH, fast low angle shot; MC-PROXYL, 3-methoxycarbonyl-2,2,5,5-tetramethylpyrrolidine-*N*-oxyl; TE, echo time; TR, repetition time. The figure was partly modified from our previous report (135).

**FIG. 5.**
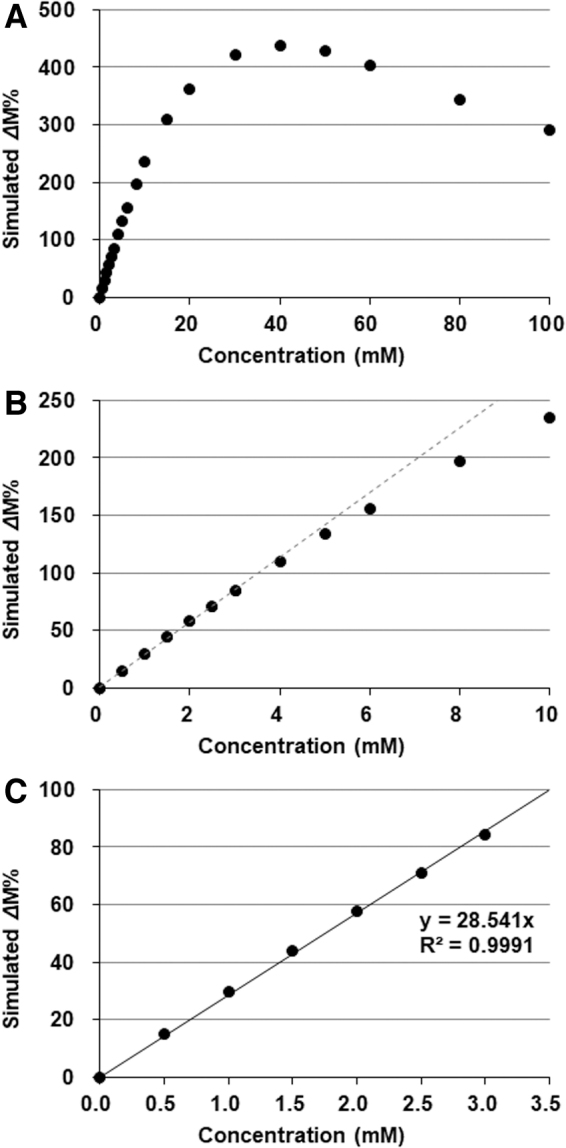
**Relationship between the concentration of nitroxyl contrast agent and simulated *Δ*M% of T_1_-weighted image. (A)** *Δ*M% increased with the concentration of paramagnetic nitroxyl contrast agent, then peaked and decreased. **(B)** The relationship between *Δ*M% and concentration of nitroxyl contrast agent slightly curved at the lower concentration region <10 m*M*. **(C)** Fairly good linearity (*R*^2^ = 0.9991) was obtained between the simulated *Δ*M% and concentration of nitroxyl contrast agent for the concentration region <3 m*M*. Values used for the simulation were as follows: r_1_ = 0.14 m*M*^−1^·s^−1^, M_0_ = 1000, TR = 75 ms, TE = 5.1 ms, FA = 45°, T_1_ of water = 2350 ms, and T_2_* = 50 ms.

The indirect detection of nitroxyl radicals based on proton MR T_1_ contrast is not quantitative. However, the low r_1_ of nitroxyl radicals gives an almost linear relationship between T_1_ enhancement and the concentration of a contrast agent in a lower concentration region (117). T_1_-weighted images of an identical phantom containing several concentrations of solutions of a nitroxyl radical, MC-PROXYL (3-methoxycarbonyl-2,2,5,5-tetramethylpyrrolidine-*N*-oxyl), scanned with the same fast low angle shot (FLASH) sequence using 1- and 7 T scanners are shown in Figure 4 (135). The r_1_ of nitroxyl radicals was estimated to be 0.27 m*M*^−1^·s^−1^ at 1 T and 0.14 m*M*^−1^·s^−1^ at 7 T, and good linearity was observed for the relationship between T_1_ enhancement and the concentration when the concentration was <3 m*M*. A plot of simulated values of T_1_-weighted gradient echo MR contrast *versus* the concentration of nitroxyl radicals calculated using a r_1_ of 0.14 m*M*^−1^·s^−1^ is shown in Figure 5. The simulation revealed an almost linear relationship between percentage signal amplification of T_1_-weighted image (*Δ*M%) and the concentration of nitroxyl contrast agent with an *R*^2^ = 0.9991 when the concentration range was <3 m*M* (Fig. 5C). Although the decay rate of *Δ*M% was slightly different from the true reduction rate of nitroxyl radicals, the differences were sufficiently small when parameters in practical ranges were used (117).

### Loading decay rate of nitroxyl radicals on the image

By attaching an additional dimension to a simple distribution image, we can obtain functional information from a set of images. The time axis is easily attached by sequential measurement of several images (118, 123). Consequently, EPR signal decay rates calculated in a pixel-wise manner, namely decay rate mapping, can be obtained.

Evidence of a faster decay rate of nitroxyl radicals in tumor tissues was obtained from EPRI experiments (101, 166). A nitroxyl radical called carbamoyl-PROXYL decayed faster in a RIF-1 tumor than in the normal tissue (101). Another study also reported that carbamoyl-PROXYL decayed faster in a SCCVII tumor than in the normal tissue (166).

**FIG. 6.**
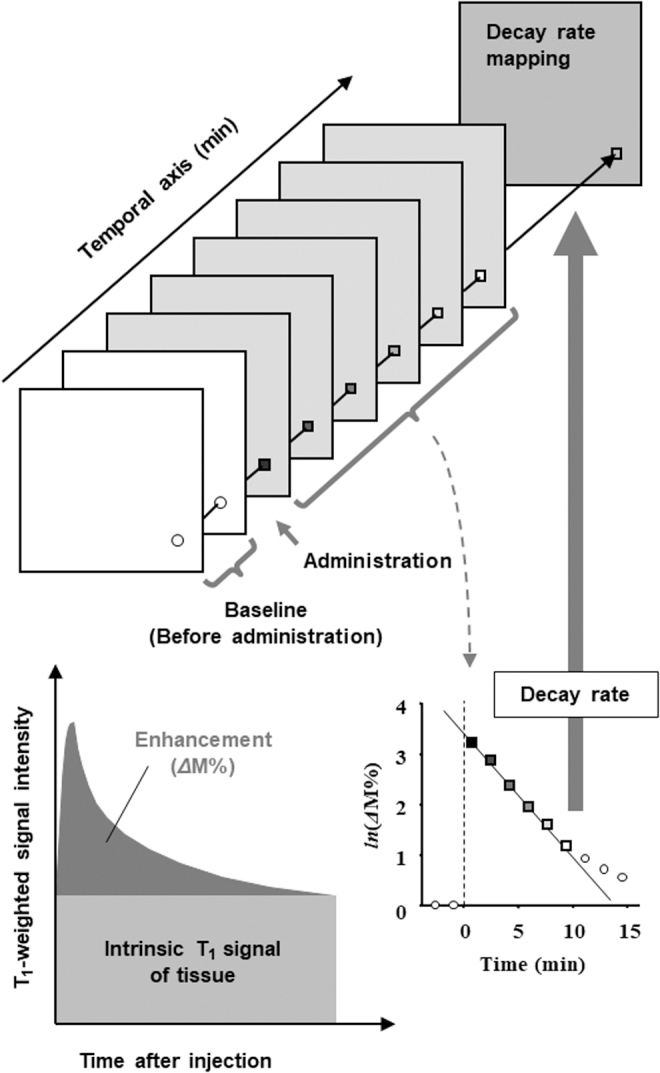
**A schematic drawing of the concept of MR redox imaging.** The additional time dimension to the 2D or 3D mapping of the contrast agent can provide functional information such as pharmacokinetics of the nitroxyl radical. The time axis can be provided by repeated scanning of an identical ROI. The *Δ*M% of the T_1_-weighted image, which is the contribution of T_1_ enhancement by nitroxyl radicals, was obtained by subtracting the baseline image from those observed after administration of the contrast agent. As a result, pixel-wise decay rates of nitroxyl radicals can be obtained. 2D, two dimensional; 3D, three dimensional; MR, magnetic resonance; ROI, region of interest.

The MR signal is composed of the intrinsic signal of the tissue and enhancement with nitroxyl contrast agent. To calculate the contribution of nitroxyl radicals to the enhanced T_1_-weighted MR signal, the baseline signal, that is, intrinsic T_1_-weighted signal of tissue before the administration of contrast agent, must be subtracted from that after the administration of contrast agent (Fig. 6). The MR signal was composed of tissue T_1_ and nitroxyl radical-induced T_1_ signals.

**FIG. 7.**
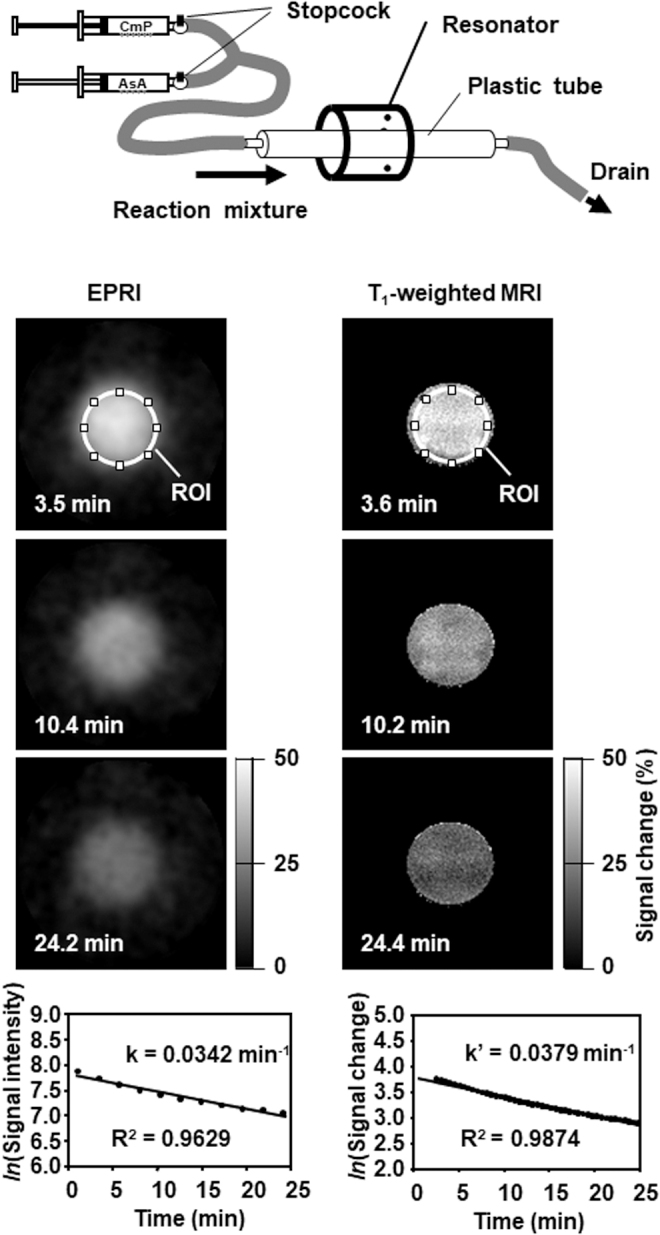
**A comparison of chemical reduction courses of a nitroxyl radical monitored by EPRI or T_1_-weighted spoiled gradient echo MRI.** (*Upper panel*) A phantom used for experiments. (*Left column* of *lower panel*) A time course of EPR images and the signal decay profile in the ROI. (*Right column* of *lower panel*) A time course of *Δ*M% signal of T_1_-weighted MRI and the signal decay profile in the ROI. An identical cylindrical phantom (internal diameter, 1.27 cm) was set in the 30 × 50 mm (diameter × length) Litz resonator operating at 300 MHz EPR or a birdcage-type MRI coil. The cylinder was previously filled with 50 m*M* phosphate buffer (pH 7.4). The same volume of 4 m*M* carbamoyl-PROXYL solution and 10 m*M* AA solution was simultaneously delivered into the cylinder with the same flow rate using a stopped-flow system; then the flow was stopped after the internal volume of the cylinder was replaced by the reaction mixture. Scans were started 5 min before the starting reaction and repeated at 2.3-min intervals for EPRI and 30-s interval for MRI. The signal decay rates were obtained from the slope of the linear portion of the decay curves. AA, ascorbic acid; carbamoyl-PROXYL, 3-carbamoyl-2,2,5,5-tetramethylpyrrolidine-*N*-oxyl; EPR, electron paramagnetic resonance; EPRI, EPR imaging; MRI, magnetic resonance imaging. The figure was partly modified from our previous report (119).

The time course of chemical reduction of a nitroxyl radical using ascorbate was compared by EPRI and T_1_-weighted MRI (Fig. 7) (119). MRI yields a higher spatial and temporal resolution than EPRI, but the resulting decay rates are similar. The decay rate obtained from MR T_1_ contrast can be handled as a proxy-decay rate, which is not the same as the original EPR decay rate, but the theoretical error between the proxy- and original EPR decay rates is sufficiently small under biological conditions (117).

### Advantages of MR detection of nitroxyl contrast agents

A relatively broad EPR linewidth and hyperfine splitting make the EPR signal intensity low in EPRI. The signal/noise ratio of EPR images decreases depending on the complication of hfs and linewidth (anisotropy) even if deconvolution techniques are used to remove spectral information (123). T_1_-weighted MRI can simplify the detection of nitroxyl contrast agents. This may be a great advantage to modify nitroxyl contrast agents chemically and to design organ/tissue specificity for *in vivo* use. The lower quantification ability of T_1_-weighted MRI may not be problematic for redox estimation. Detection of nitroxyl by T_1_-weighted MRI is fast and provides high resolution.

Coupling multiple nitroxyl molecules can improve the T_1_-weighted contrast effects of nitroxyl-based contrast agents (124). A good linear relationship was obtained between T_1_-weighted image enhancement and the concentration of the contrast agent up to three spins in a molecule. The r_1_ levels of nitroxyl contrast agents increase depending on the number of nitroxyl spins in a molecule. Although the coupling of multiple nitroxyl molecules broadens its EPR spectrum and makes EPRI difficult, T_1_-weighted MRI techniques enable the mapping of the multispin nitroxyl contrast agents. However, support by EPR spectroscopy remains important to understand information from MRI.

**FIG. 8.**
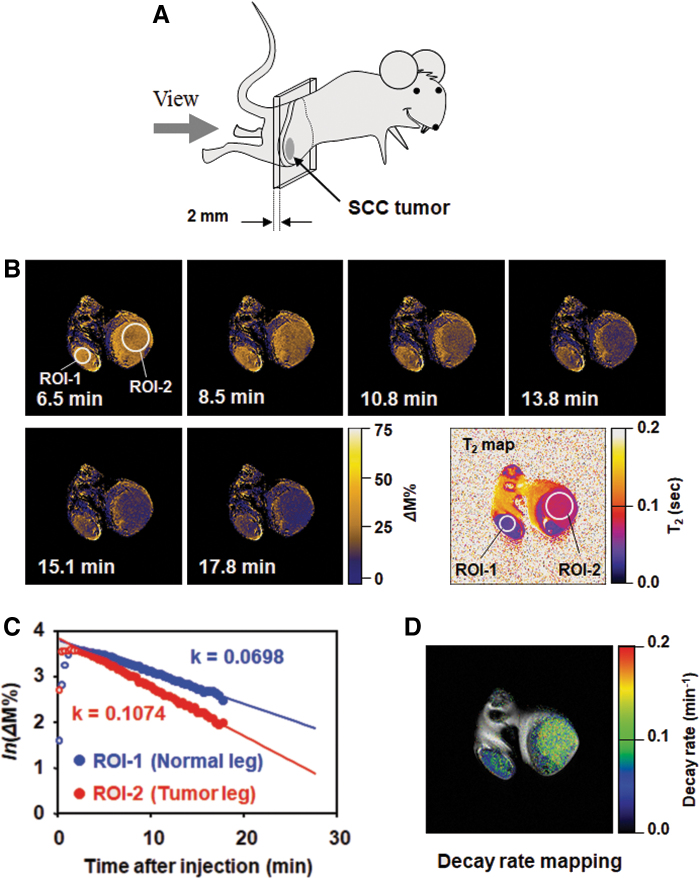
**An example of MR-based redox imaging. (A)** Direction of the slice view of MRI with respect to the subject mouse. **(B)** Time course of *Δ*M% signal of T_1_-weighted MRI and scout T_2_ mapping for ROI selection. Time after injection is indicated in each image. ROI-1 for normal leg and ROI-2 for tumor leg were estimated based on previous T_2_ mapping. The field of view was 3.2 × 3.2 cm. **(C)** Time course of average *Δ*M% signal in ROI-1 and ROI-2. Logarithmic values of *Δ*M% signal in the ROIs are plotted against time. Decay rate constants were obtained from the slope of linear decay after the peak. **(D)** The decay rate map overlapped on the corresponding multislice multiecho image shows the distribution of decay rates with clear anatomic information. The figure was partly modified from our previous report (119). Color images are available online.

Mapping the *in vivo* nitroxyl decay rate in a SCCVII tumor on the mouse thigh was measured using MRI (119). The location of the axial slices including the SCCVII tumor and normal leg of a mouse is shown in Figure 8A. T_1_ contrast was enhanced in both normal and tumor tissues after the administration of carbamoyl-PROXYL, and then it gradually decreased (Fig. 8B). The time course of T_1_ contrast in the region of interest (ROI)-1 and ROI-2 is shown in Figure 8C. The tumor region exhibited a faster decay rate than the normal region. The decay rate mapping (Fig. 8D) demonstrated a notable difference in decay rates between tumor tissue and the normal tissue. The difference between tumor tissue and normal tissue remained around the tumor tissues and was clear due to the high spatial resolution.

### Redox information rather than clearance

**FIG. 9.**
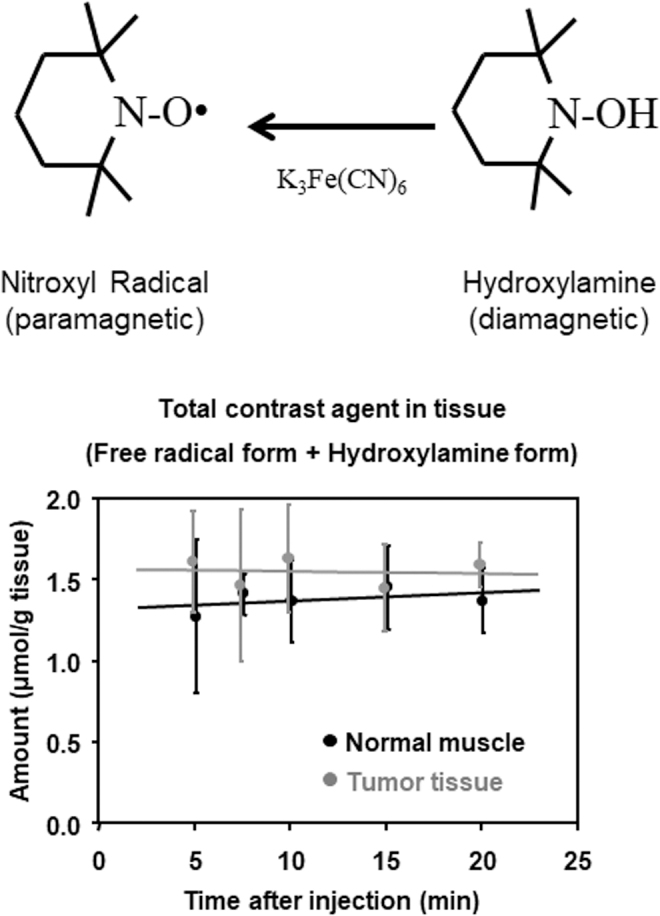
**Time course of total (nitroxyl radical form+hydroxylamine form) amount of carbamoyl-PROXYL in the normal muscle (*black circle*) and tumor tissues (*gray circle*) after the i.v. injection of the nitroxyl radical form of carbamoyl-PROXYL.** Each tissue from each mouse was homogenized, and then K_3_[Fe(CN)_6_] was added to the homogenate to make the final concentration 2 m*M* for oxidizing the hydroxylamine form to the nitroxyl radical form. The homogenate from each mouse was measured by X-band EPR in triplicate. Marks and error bar indicate the average ± SD of three mice. The *horizontal lines* in the figure are obtained by least-squares fitting for the values of normal and tumor tissues. SD, standard deviation. The figure was partly modified from our previous report (119).

Hydroxylamine can be easily oxidized to the corresponding nitroxyl radical by adding a strong oxidant such as potassium ferricyanide. Then, the total amount of contrast agent in the tissue, including the nitroxyl radical form plus hydroxylamine form, can be measured by EPR. The time course of the total amount of contrast agent in both the tumor and normal tissues was stable during the time period used in the imaging experiment (Fig. 9). Therefore, the *in vivo* disappearance of EPR signal and T_1_ contrast induced by a nitroxyl contrast agent is due to reduction (119).

**FIG. 10.**
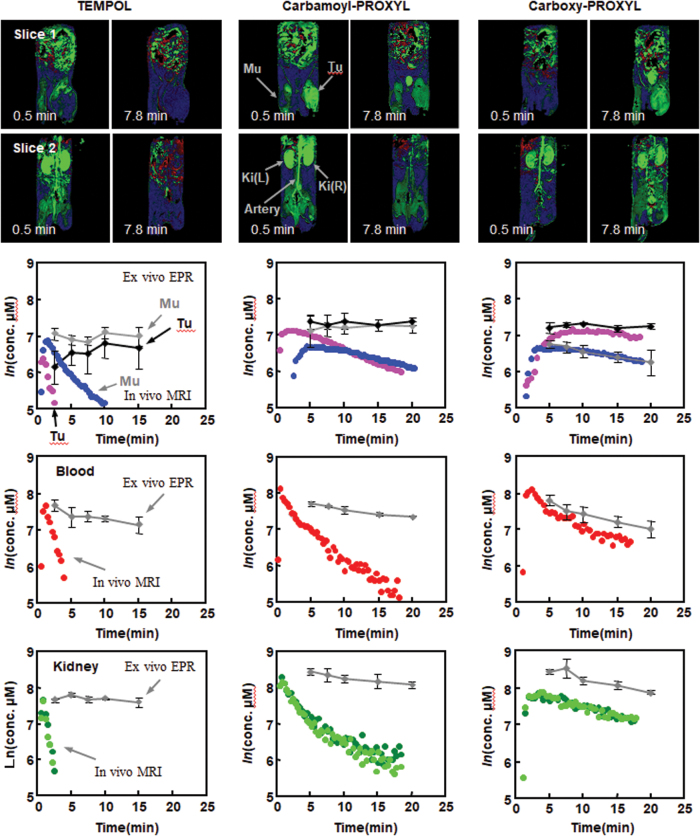
**Comparison of pharmacokinetic profiles of three nitroxyl contrast agents by T_1_-weighted MRI.** (*Upper image panels*, Slice 1) *Δ*M% images of a 2 mm-thick slice containing the normal left leg (Mu) and tumor planted on the right leg (Tu) scanned after the administration of TEMOPL (*left*), carbamoyl-PROXYL (*center*), or carboxy-PROXYL (*right*). The time after injection is indicated in each image. (*Lower image panels*, Slice 2) *Δ*M% images of a 2 mm-thick slice containing the artery and the right and left kidneys, Ki(R) and Ki(L) simultaneously obtained for the same mouse in Slice 1. (*Lower panels*) The pharmacokinetic profiles of the oxidized form and total (nitroxyl radical form+hydroxylamine form) TEMPOL (*left*), carbamoyl-PROXYL (*center*), and carboxy-PROXYL (*right*). The time course of nitroxyl radical form in normal tissue (*blue circle*), tumor tissue (*purple circle*), blood (*red circle*), and kidney (left kidney, *dark green circle*; right kidney, *light green circle*) were obtained by T_1_-weighted MRI. The concentrations of total nitroxyl contrast agent (nitroxyl radical+hydroxylamine) measured by X-band EPR spectroscopy in the corresponding tissues are indicated by *gray diamonds* or *black diamonds* for tumor tissue. carboxy-PROXYL, 3-carboxy-2,2,5,5-tetramethylpyrrolidine-*N*-oxyl; TEMPOL, 4-hydroxy-2,2,6,6-tetramethylpiperidine-*N*-oxyl. The figure was partly modified from our previous report (79). Color images are available online.

The pharmacokinetics of three different nitroxyl contrast agents with different membrane permeability were investigated (79). TEMPOL is an amphiphilic molecule with high membrane permeability. Carbamoyl-PROXYL has slight membrane permeability. However, carboxy-PROXYL (3-carboxy-2,2,5,5-tetramethylpyrrolidine-*N*-oxyl) is membrane impermeable. T_1_-weighted signal amplification (*Δ*M%) images and pharmacokinetic profiles after the administration of nitroxyl contrast agents are shown in Figure 10. The concentration of the free radical forms of nitroxyl contrast agents in the tissue calculated based on the enhancement of T_1_ contrast and the concentration of the total amount of contrast agents according to EPR measurement were compared (Fig. 10, lower panels). For TEMPOL and carbamoyl-PROXYL, T_1_ contrast decay curves demonstrated faster decay than that of the total contrast agents. Carboxy-PROXYL, however, exhibited similar decay slopes between T_1_ contrast and the total contrast agent. Cell-impermeable nitroxyl radicals are stable against reduction. Redox imaging techniques using a combination of a membrane-permeable nitroxyl radical and dynamic scanning T_1_-weighted MRI can provide redox information for the target tissue.

### Approaches of redox imaging to planning radiation therapy

Ionizing radiation ionizes or excites water molecules, and then generates ^•^OH, hydrogen radicals (^•^H), hydrated electrons (e^−^_aq_), and other ions (129). Due to its markedly high reactivity, ^•^OH is generally considered to be the major oxidative attacker in radiation biology. Molecular O_2_ can react with ^•^H to make a HO_2_^•^ and superoxide (O_2_^•−^). Hydrogen peroxide (H_2_O_2_) can be generated by the reaction of 2 HO_2_^•^ or reaction of 2 ^•^OH. As 70%–80% of the body volume is composed of water molecules, 70%–80% of the effects of ionizing radiation are due to indirect actions, in which directly generated reactive species by water radiolysis or secondary generated species attack biological molecules. Therefore, most of the effects of radiation are due to the oxidative stress by such reactive oxygen species (ROS).

The results of radiation therapy would be affected by physiological and/or pathological conditions in the target tissues, especially by the low partial oxygen pressure (pO_2_) in cancer/tumor tissues. The tissue pO_2_ affects the yield of secondary ROS such as H_2_O_2_, O_2_^•−^, and/or other reactive species in the tissue (122). Therefore, the tissue pO_2_ and/or accompanying tissue redox status is important for the effectiveness of radiation therapy. For safer and more accurate cancer/tumor radiation therapy, a diagnostic method for tissue pO_2_ and/or tissue redox status using a noninvasive functional imaging technique is required (123, 166).

**FIG. 11.**
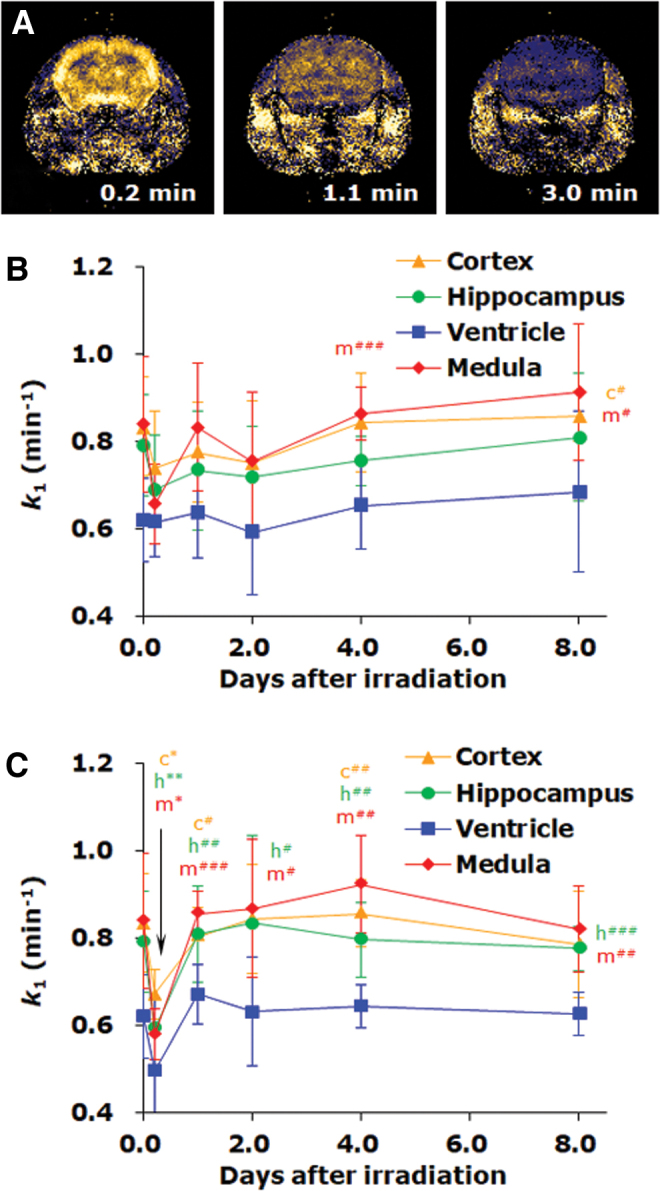
**The time course of redox status in the mouse brain after irradiation by X-rays or carbon-ion-beams. (A)** Distribution of a nitroxyl contrast agent, MC-PROXYL, in mouse brain and the time course of *Δ*M%. **(B)** Responses of the *k*_1_ decay rate of MC-PROXYL in the mouse brain after 8 Gy X-ray irradiation for a week. **(C)** Responses of pharmacokinetic parameters of MC-PROXYL in the mouse brain after 8-Gy carbon-ion-beam irradiation (290 MeV mono beam, LET = 60 keV/μm at the surface) for a week. The values on the *y*-axis (0 day) indicate those for normal healthy mice (*n* = 12). Other values are indicated by the average ± SD of nine mice for X-rays and of six mice for carbon ion beams, except for the values several hours (0.2 day) after irradiation, which were averaged from three mice. The figure was partly modified from our previous report (136). The letters indicate the initial character of corresponding brain region, and single, double, and triple asterisk(s) or #(s) indicate grades of significance, i.e. *p* < 0.05, *p* < 0.01, and *p* < 0.001, respectively. Color images are available online.

The time course of redox status in the mouse brain after irradiation by X-rays or carbon-ion-beams was investigated (136). The signal decay rate *k*_1_ decreased several hours after irradiation, and then gradually recovered to the original level after X-ray irradiation; however, it mostly recovered or exceeded the original level 1 day after carbon-beam irradiation (Fig. 11). The decrease in *k*_1_ observed several hours after irradiation may be due to reduced blood flow because both decay rates at the later time window *k*_2_, which reflects clearance rather than reduction of nitroxyl radicals, and maximum signal intensity temporally decreased several hours after irradiation. The brain exhibited different responses to X-rays and carbon beams. Ionizing radiation can affect the brain tissue redox status for a week, and redox imaging can visualize the tissue status that was not visually discernable.

The biological effects of radiation persist, causing symptoms at a late stage even if there were no visible symptoms at an early stage after irradiation. The prognosis and prevention of late-onset disorders of radiation can be investigated using redox imaging.

### Approaches of MR redox imaging for theranostic applications

**FIG. 12.**
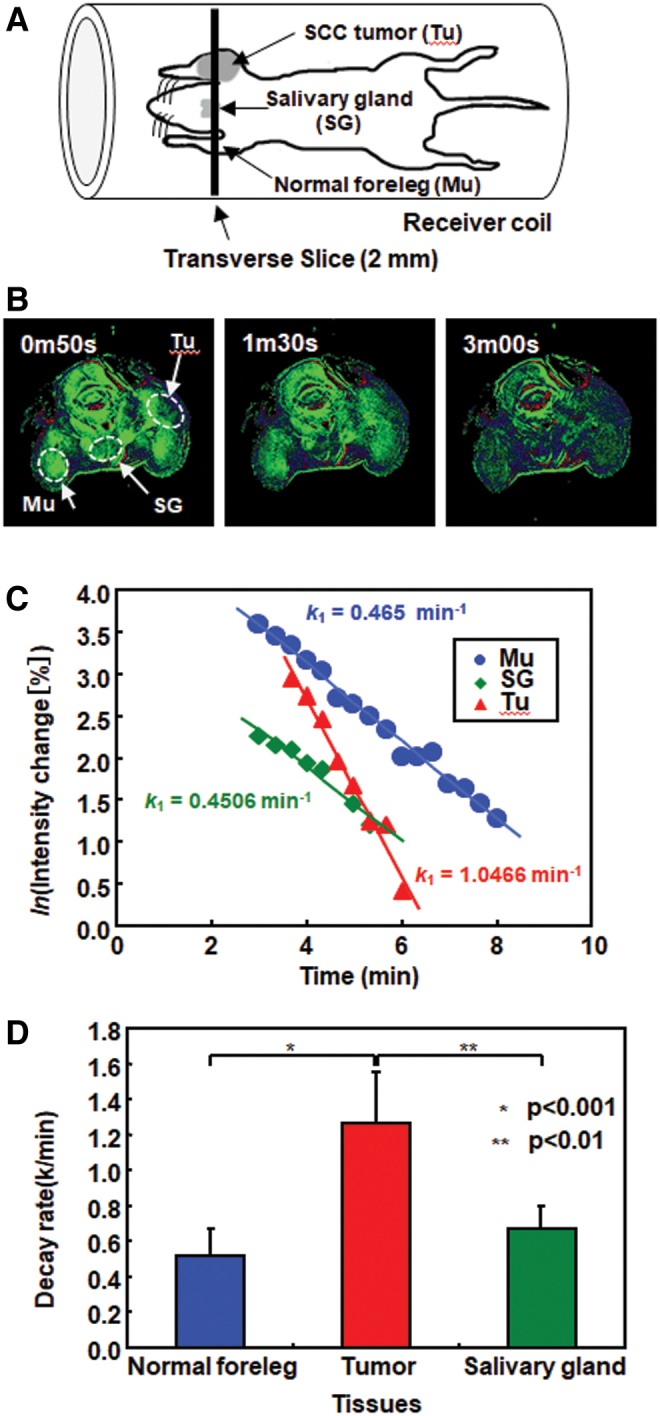
**Comparison of decay rates of TEMPOL in tumor and normal tissues. (A)** A schematic drawing of the position of the mouse in the resonator and the slice selected for MRI experiments. A transverse slice (2 mm) covering the normal muscle tissue on foreleg (Mu), SG, and the tumor in the contralateral leg (Tu) was selected to monitor the time course of TEMPOL-induced signal. **(B)** T_1_-weighted images of the selected region after i.v. injection of TEMPOL. The ROIs were selected in the normal leg, SG, and tumor to monitor TEMPOL decay rates. **(C)** Representative TEMPOL decay profiles after i.v. injection in a mouse for the selected ROIs. **(D)** Summary of decay rates from the three ROIs in normal muscle, SG, and tumor (*n* = 4 for SG and *n* = 6 for tumor per normal leg). The figure was partly modified from our previous report (20). Color images are available online.

The mechanism of the radioprotection effects of TEMPOL on the salivary gland was analyzed using MR redox imaging (20). A SCCVII tumor was prepared on the right front leg of mice to obtain the salivary gland and tumor tissue in an identical slice (Fig. 12A). Increased T_1_-weighted MR signal induced by TEMPOL quickly appeared and peaked at 1 min, and then gradually disappeared (Fig. 12B). Natural logarithmic values of TEMPOL-mediated MR intensity in normal muscle, tumor, and salivary gland were plotted as a function of time after the injection of TEMPOL, and then decay rates were obtained from the slopes (Fig. 12C). The decay rate in MR intensity was similar for normal leg tissue and the salivary gland; however, the decay rate was significantly faster in the tumor (Fig. 12D). The differential radioprotection by TEMPOL resides in the faster reduction to the nonradioprotective hydroxylamine in the tumor than in normal tissues.

**FIG. 13.**
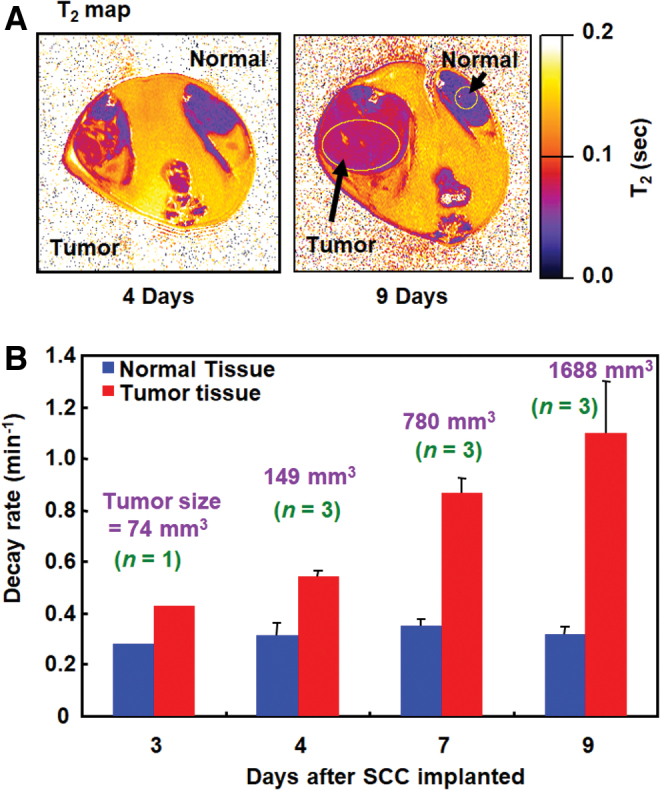
**Relationship between TEMPOL reduction rate and tumor size. (A)** T_2_ map of the SCCVII tumor grown on the hind leg of the same mouse scanned at days 4 and 9. **(B)** Decay rates of TEMPOL in normal muscle and tumor, and the tumor size as a function of time. The figure presents data from experiments in our previous report (78). Color images are available online.

The hypoxic environment in tumor tissues inhibits reoxidation of a hydroxylamine to the corresponding nitroxyl radical, and the apparent reduction rate consequently markedly increases. Hyodo *et al.* (78) reported that the reduction rate of a nitroxyl radical increased as a function of tumor size (Fig. 13). The markedly higher reduction rates of nitroxyl contrast agents in tumors than in normal tissues can be exploited for diagnosis.

Different redox environment between normal and tumor tissues causes the difference in concentration of nitroxyl radicals between normal and tumor tissues after administration. The concentration of the free radical form in the tumor tissue rapidly decreases and remains around zero; however, that in the normal tissue remains slightly higher due to reoxidation of the hydroxylamine form. The nitroxyl contrast agents can be normal tissue-selective radioprotectors in radiation therapy, which may be carried out after the diagnosis. The nitroxyl contrast agents can be used twice during radiation therapy, first as a redox probe and second as a radioprotector for normal tissues.

**FIG. 14.**
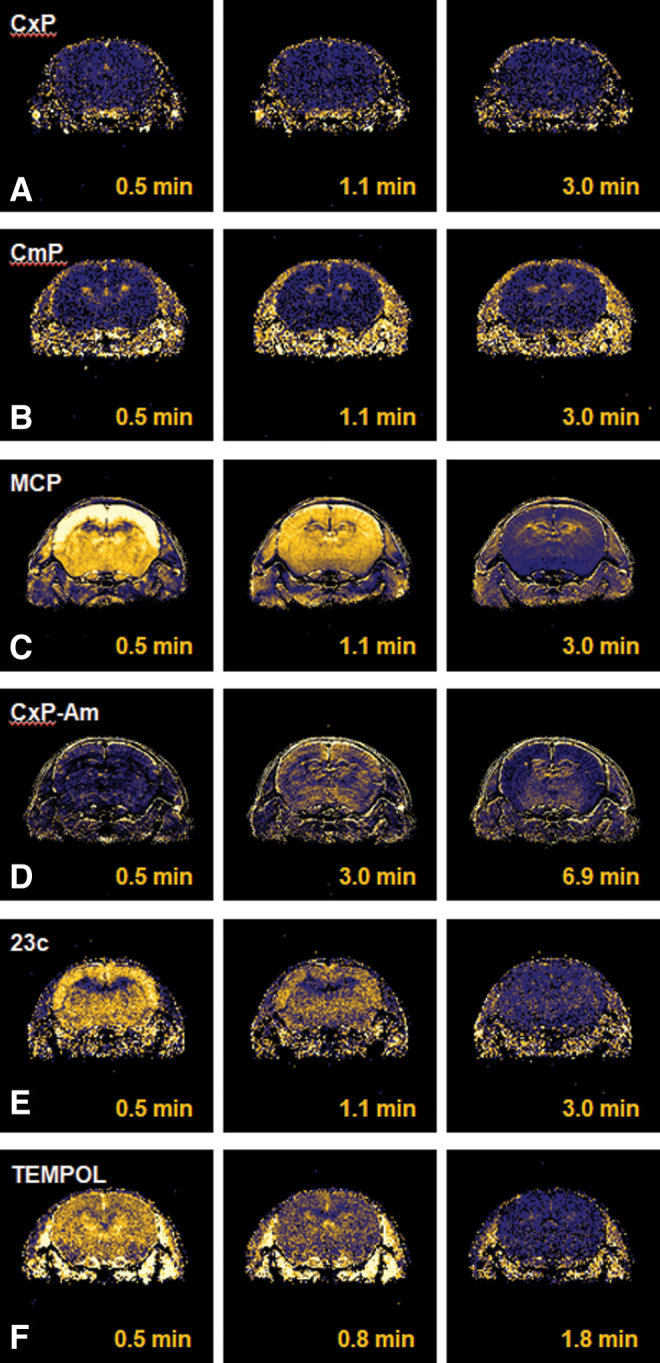
**Distributions of nitroxyl contrast agents in the mouse brain.** T_1-_weighted signal enhancement in the mouse head was observed after i.v. injection of **(A)** carboxy-PROXYL, **(B)** carbamoyl-PROXYL, **(C)** MC-PROXYL, **(D)** CxP-Am, **(E)** 23c, or **(F)** TEMPOL. The *horizontal row* shows the time course of the T_1_-weighted signal enhancement. 23c, 4-(*N*-methylpiperidine)-2,2,5,5-tetramethylpyrroline-*N*-oxyl; CxP-Am, acetoxymethyl-2,2,5,5-tetramethyl-pyrrolidine-*N*-oxyl-3-carboxylate. The figure was partly modified from our previous report (125) with some additional data. Color images are available online.

Another future possibility of nitroxyl contrast agents is in brain imaging (23, 118, 125). There are several nitroxyl radicals with the ability to permeate the cell membrane because their membrane permeability can be easily regulated by altering a part of the molecule. Nitroxyl-induced T_1_ contrast in the mouse head is shown in Figure 14. Different distributions of nitroxyl contrast agents in the brain are observed depending on blood–brain barrier (BBB) permeability. Membrane-impermeable carboxy-PROXYL resulted in no T_1_ contrast in the brain (Fig. 14A), whereas carbamoyl-PROXYL, which has slight membrane permeability, partly induced T_1_ contrast in the brain (Fig. 14B). Highly permeable MC-PROXYL, 23c [4-(*N*-methylpiperidine)-2,2,5,5-tetramethylpyrroline-*N*-oxyl] (23), and TEMPOL demonstrated high T_1_ contrast induction in the entire brain (Fig. 14C, E, F). CxP-Am (acetoxymethyl-2,2,5,5-tetramethyl-pyrrolidine-*N*-oxyl-3-carboxylate), which is a BBB-permeable molecule, was hydrolyzed to membrane-impermeable carboxy-PROXYL in the brain and remained there for a long time (Fig. 14D) (157). The structures of BBB-permeable nitroxyl probes are shown in Supplementary Figure S1.

Investigations of *in situ* or *in vivo* drug delivery using nitroxyl-labeled drugs have long been conducted in the field of EPRI (39, 40, 45). In MRI, Zhelev *et al.* (196) successfully visualized that i.v.-injected TEMPO-labeled nitrosourea (SLENU), which is an anticancer drug labeled by a nitroxyl radical, could penetrate through BBB and distribute into whole brain. This is to say a redox-sensitive paramagnetic contrast agent has a medicinal benefit and vice versa. Using such a hybrid contrast agent, the distribution of the drug and additionally tissue redox status could be observed.

In addition, nitroxyl radicals itself have preventive effects against inflammation, and the mechanisms and applications have been investigated. Deguchi *et al.* (24) reported preventive effects of nitroxyl radical compounds against indomethacin-induced gastric ulcers in rats based on OMRI signal decay. Eguchi *et al.* (37) reported that daily gavage of polymeric micelles possessing nitroxyl radicals for 4 weeks decreases hepatic inflammation in nonalcoholic steatohepatitis model mice. Diagnostic processes of redox status in an inflammatory tissue by a redox imaging with a suitable nitroxyl contrast agent may also be a therapeutic process.

Another design of nitroxyl radical-labeled polymer-type contrast agents, which have dendrimer core and lapping polyethylene glycol chains, was proposed for MRI (139). A nitroxyl radical, fluorophore, prodrug was loaded onto the lapping polymer, and the polymers were conjugated on the core. This structured polymer contrast agent simultaneously works as a drug carrier.

## Nitroxyl Radical Contrast Agent for Imaging Redox Imbalance and Oxidative Stress in Living Biological Subjects

### Imaging redox imbalance and oxidative stress in the brain

Due to its complex structure and functions, brain is one of the major targets for redox imaging using nitroxyl-enhanced EPRI and MRI, especially at aging, immobilization stress, neurodegenerative damage, hypoxia, and others (156, 179).

**FIG. 15.**
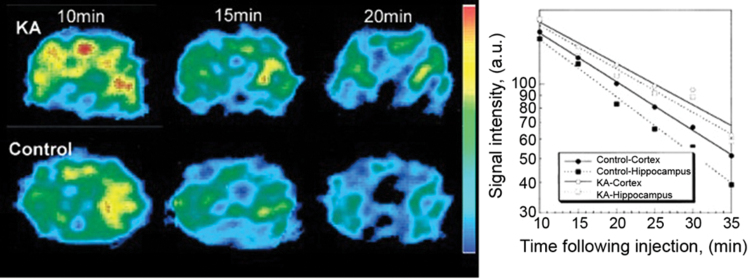
**EPRI of the rat brain.** *Left*: The dynamic pattern of selected transversal EPR images of the rat head 5 mm posterior to the bregma in the KA-treated and control groups at different times after injection of nitroxyl radicals (MC-PROXYL). *Right*: Pharmacokinetic curves for brain regions. The cortical half-lives of MC-PROXYL in the control and KA groups were 18.0 ± 1.2 and 19.2 ± 0.7 min, respectively, whereas the hippocampal half-lives of MC-PROXYL in the control and KA groups were 10.4 ± 0.8 and 15.9 ± 0.7 min, respectively. [Adapted from Yokoyama *et al.* (190).] KA, kainic acid. Color images are available online.

Yokoyama *et al.* published a series of studies on EPRI of brain injuries induced in experimental animals accompanied by the development of oxidative stress (187–191). The authors' basic concept of time-resolved EPRI of the brain is presented in Figure 15 (190). In the brains of rats with seizures induced by kainic acid, the half-life of the EPR signal of the nitroxyl radical MC-PROXYL was longer in the hippocampus than in the cortex, indicating a lower reducing ability of the hippocampus.

These observations were confirmed by the injection of diamagnetic acyl-protected hydroxylamine ACP, which undergoes intracellular oxidation to a nitroxyl radical (188). In this case, the authors detected EPR signal and an increase in its intensity in the hippocampus and striatum of kainic acid-treated animals, but not in the cortex. In other studies, the same authors treated animals with neuroleptics known to induce oxidative stress in the brain and analyzed the tissue redox status using MC-PROXYL (187, 189, 191). The brain tissues exhibited lower reducing capacity of the injected nitroxyl probe. This decrease in reducing capacity was also observed in elderly rats compared with young individuals kept under standard conditions (61, 140, 191).

One of the most widely used models for the induction of oxidative stress in the brain is experimental ischemic–reperfusion injury caused by reversible occlusion of the carotid artery (“middle cerebral artery occlusion model”). This condition is accompanied by the generation of high amounts of ROS and induction of oxidative stress in the affected areas (148). Lower reducing capacity was also reported in this model using MC-PROXYL as a redox-sensitive contrast probe and nitroxyl-enhanced EPRI and MRI (77). Similar results were reported by Yokoyama *et al.* (187) in an EPRI study of a rat model of neonatal ischemia-induced reperfusion encephalopathy, using 3-(hydroxymethyl)-1-oxy-2,2,5,5-tetramethylpyrrolidine (HM-PROXYL). Treating animals with an antioxidant abolishes (removes) this effect.

Another approach to alter the tissue redox status is the induction of septic shock by i.v. injection of lipopolysaccharides (LPS) in animals. In this case, EPRI revealed that the rate and degree of reduction of the HM-PROXYL spin probe increased in all parts of the brain of LPS-treated mice (47). Pretreatment of mice with allopurinol (xanthine oxidase inhibitor) or aminoguanidine (NOS inhibitor) suppressed the effects of septic shock on the degree of HM-PROXYL reduction, which is indirect evidence of the role of O_2_^•−^ and nitric oxide in the dynamics of nitroxyl contrast.

**FIG. 16.**
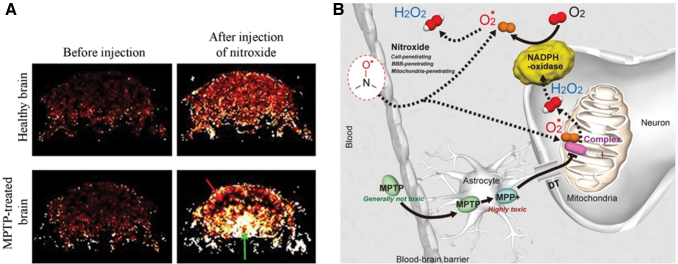
**Visualization of superoxide production *in vivo* in the dopaminergic area of the brain in Parkinson’s disease. (A)** Nitroxyl-enhanced MR imaging of tissue redox status in dopaminergic neurons [according to Zhelev *et al.* (195)]. Extracted nitroxyl-enhanced MRI signal in the brain of healthy and MPTP-treated mice obtained 5 min after the injection of nitroxyl contrast agent (mito-TEMPO; T_1_-weighted MRI, gradient-echo). The *green arrow* indicates the *SNpc*. The *red arrow* indicates the cortex. **(B)** Molecular hypothesis for enhancement of MRI signal in MPTP-affected and oxidatively active dopaminergic neurons [according to Vila and Przedborski (182) and Zhelev *et al.* (195)]. DT, dopamine transporter; mito-TEMPO, 2,2,6,6-tetramethyl-4[[2-(triphenylphosphonio)acetyl]amino]-1-piperidinyloxy; MPP^+^, 1-methyl-4-phenylpyridinium; MPTP, 1-methyl-4-phenyl-1,2,3,6-tetrahydropyridine. Color images are available online.

In 2013, a methodology for direct visualization of O_2_^•−^ production *in vivo* in the dopaminergic area of the brain in Parkinson's disease was developed, based on the redox cycle of mito-TEMPO (2,2,6,6-tetramethyl-4[[2-(triphenylphosphonio)acetyl]amino]-1-piperidinyloxy) and its MRI contrast (195). The experiments were conducted on healthy and 1-methyl-4-phenyl-1,2,3,6-tetrahydropyridine (MPTP)-treated mice (Fig. 16). In healthy mice, the nitroxyl-enhanced MRI signal in the “substantia nigra pars compacta” (*SNpc*) was weak and short lived. The histograms suggested high reducing activity of normal brain tissues against mito-TEMPO. In MPTP-treated mice, the nitroxyl-enhanced MRI signal in the *SNpc* was strong and long lived. The histograms suggested high oxidative activity of dopaminergic tissues in the MPTP-treated brain. This nitroxyl-based MRI study demonstrated that O_2_^•−^ is a major inducer and/or mediator of neurodegenerative damage in Parkinson's disease in mammals.

### Imaging redox imbalance and oxidative stress in cancer

It is generally accepted that the redox signaling of cancer cells and tissues is different from that of normal tissues due to the hypoxic environment, increased levels of ROS (especially O_2_^•−^), and increased amount of reducing equivalents (mainly glutathione) in tumors (16, 83, 93, 97, 143, 174, 175). The effectiveness of radiation therapy and many chemotherapeutic approaches depends on the degree of oxygenation in tumors (19, 51). A number of studies on cancer models have been published (22, 79–81, 101, 102, 119, 168).

Imaging is the preferred technique for functional diagnostics of cancer due to the heterogeneity of tumor tissues in two aspects—their redox status and oxygenation (4, 22, 175). Therefore, spatially separated images for both are preferred: (i) the distribution of nitroxyl contrast agent in the tumor and its elimination from tumor tissue; and (ii) tumor oxygenation. To achieve this goal, different approaches are used by combining EPR and MRI, and the selection of appropriate contrast probes (50, 54, 79–81, 101, 102, 127, 147, 168, 183).

An early example of visualization and analysis of redox status of tumors in mice by nitroxyl-enhanced MRI and EPRI using carbamoyl-PROXYL as a redox-sensitive contrast agent is presented in Figure 8 (119). The authors of this study suggested that the rate of reduction of the nitroxyl radical carbamoyl-PROXYL in tumor tissue is higher than that in nontumor tissues based on the increased levels of endogenous reducing equivalents in tumor cells (mainly glutathione and glutathione-dependent antioxidants), in addition to the presence of hypoxia in tumors. Similar opinions have been expressed by other authors using carboxy-PROXYL or carbamoyl-PROXYL as contrast probes (79–81, 101, 102, 127, 183).

*In vivo* studies in tumor-bearing animals using ^13^C-dehydroascorbate as an endogenous redox sensor and hyperpolarized MRI revealed that tumor tissues are characterized by higher levels of glutathione and ascorbate than normal tissues of healthy animals (90, 91). However, the authors noted that the ratio of oxidized/reduced forms of the two substances is in favor of the oxidized form in the tumor tissue and in favor of the reduced form in the normal tissues. This suggested that the reducing capacity of normal (healthy) tissues is higher than that of tumor tissues.

Roshchupkina *et al.* (150) reported a unique approach to determine the amount of reduced glutathione in isolated cancer cells and tissues by EPR spectroscopy, using two ^15^N-labeled nitroxyl rings connected by a disulfide bridge. The EPR spectrum of the biradical differs from that of the monomer nitroxyl radical, which enables monitoring of the reaction of the probe with reduced glutathione.

**FIG. 17.**
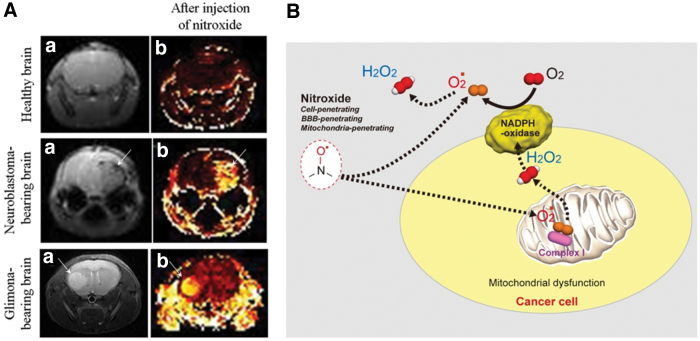
**Nitroxyl radicals as redox sensors to visualize and analyze the tissue redox status in cancer tissues. (A)** Typical MR images of healthy brain and cancer-bearing brain of mice: **(A.a)** MR images of the mouse brain; **(A.b)** extracted nitroxyl-enhanced MRI signal obtained 6 min after the injection of nitroxyl radicals (SLENU). The *arrows* indicate the tumor area. **(B)** Molecular hypothesis for increased MRI signal in metabolically (oxidatively) active cancer cells and tissues. SLENU, TEMPO-labeled nitrosourea [according to Zhelev *et al.* (194) and Bakalova *et al.* (6)]. Color images are available online.

In our recent studies, we used several nitroxyl radicals as redox sensors to visualize and analyze the tissue redox status in cancer tissues *in vivo* using MRI on animals: (i) SLENU—highly hydrophobic, cell-penetrating, and DNA-alkylating; (ii) TEMPOL—amphiphilic, cell-penetrating; and (iii) carbamoyl-PROXYL—hydrophilic and very low/nonpenetrating in living cells and tissues (6, 194, 197, 198). A strong and long-lived nitroxyl-enhanced MRI signal was detected in the cancer tissues, and a relatively strong and long-lived signal was detected in the surrounding tissues of cancer-bearing mice (Fig. 17). In contrast, a short-lived nitroxyl-enhanced MRI signal was detected in the tissues of healthy mice. This suggested that cancer and noncancer tissues of cancer-bearing animals are characterized by high oxidative activity toward nitroxyl radicals, whereas tissues of healthy animals are characterized by high reducing activity. It should be noted that SLENU was the most appropriate nitroxyl probe to evaluate tissue redox status *in vivo* due to its easy intracellular delivery and prolonged retention in the tissues. In addition, our recent EPR study on cultured cells with different proliferative indexes demonstrated that EPR signal decay is well correlated with proliferating activity (199). The slowest rate of EPR signal attenuation was detected in rapidly proliferating cancer cells, with a higher rate in slowly proliferating noncancer cells and the highest rate in nonproliferating cells.

### Imaging redox imbalance and oxidative stress in the kidneys

Hirayama *et al.* (73) used mice with acute renal failure due to ischemia–reperfusion and noted a slower decrease in EPR signal of carbamoyl-PROXYL than that in controls. This result can be explained by the induction of oxidative stress in the kidneys of mice with impaired renal function. The same group also published an EPRI study of the redox status of renal tissues in mice with hypertension induced by ligation of the right renal artery (74). In mice with hypertension, the half-life of the nitroxyl-enhanced EPR signal was longer than that in healthy mice. Treatment of mice with azelnidipine (a calcium channel blocker) for 2 weeks improved the reducing ability of the kidneys and shortened the life of the nitroxyl radicals. The same group also used another model of renal dysfunction, Nrf2-deficient mice with lupus-like autoimmune nephritis (75). The fourfold longer half-life of EPR signal of carbamoyl-PROXYL in the injured tissues of Nrf2-deficient elder mice compared with that of young wild-type mice was due to the combination of transcription factor deficiency and aging. The authors suggested that a lower reducing ability plays a role in the development of autoimmune nephritis.

Carbamoyl-PROXYL and EPRI were also applied to redox imaging of the kidneys of a murine model of streptozotocin-induced diabetes (120, 158, 161, 162, 176). These studies reported faster reduction of the nitroxyl probe in the kidneys of diabetic mice. It is generally accepted that diabetes is accompanied by the development of oxidative stress in tissues, with ROS playing a significant role in the pathogenesis of the disease (88, 149). However, it is unclear why oxidative stress in the kidneys leads to a delay in some cases (73–75, 161) and to acceleration of the EPR signal attenuation of the nitroxyl probe in others (120, 158, 161, 162, 176).

Brasch *et al*. (14) investigated the dynamics of nitroxyl-enhanced MRI in the healthy animals and animals with experimental renal ischemia and hydronephritis, using an amphiphilic nitroxyl radical 4-[(3-carboxy-1-oxopropyl)amino]-2,2,6,6-tetramethyl-1-piperidinyloxy (TES). Increased contrast was found in damaged kidneys compared with kidneys in healthy animals. Renal ischemia and hydronephritis are accompanied by the induction of high oxidative stress in the kidneys due to mitochondrial dysfunction, and production of inflammatory factors and ROS/reactive nitrogen species (7, 92, 110, 151, 152, 165). Decreased perfusion in damaged kidneys has also been reported in many studies (7, 116), which implies that penetration of this organ by contrast agent is difficult. Therefore, the increased contrast of TES in the damaged kidney after ischemia/reperfusion is most likely a result of oxidative stress and existence of nitroxyl contrast agent mainly in a radical form.

Although a possibility of delayed or accelerated filtration of nitroxyl contrast agent in the kidneys would be the main factors that can affect the dynamics of the nitroxyl probes in this organ regardless of the redox state of the tissues, discussion about renal clearance of the past experimental model had been lacking. In models of renal dysfunction, at least one conventional contrast agent and technique, such as gadolinium-enhanced MRI, must be used to determine whether the signal dynamics in the damaged kidney are the result of an increased or decreased filtration rate.

**FIG. 18.**
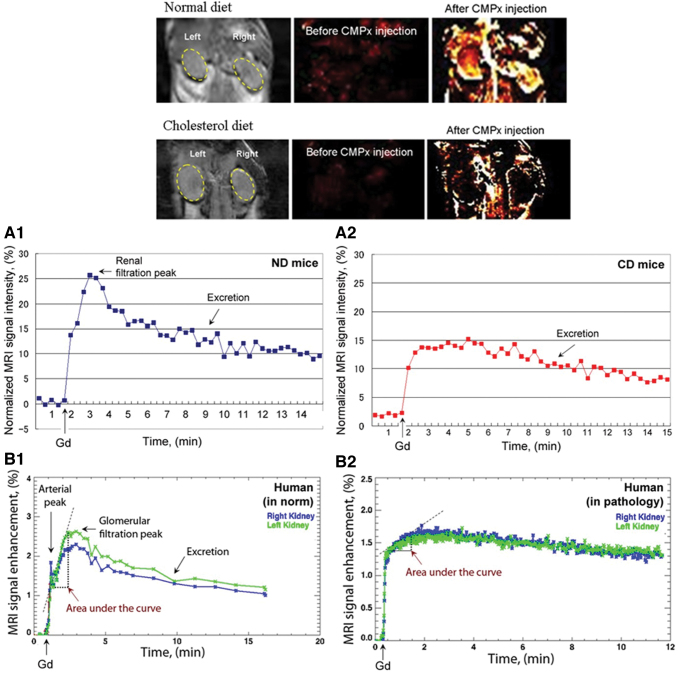
**Nitroxyl-enhanced MRI of the kidney in mice on a ND or high CD using carbamoyl-PROXYL as a contrast probe: black and white images: T_1_-weighted MR images of the kidney before the injection of carbamoyl-PROXYL.** Color images: extracted MRI signal intensity normalized to the averaged baseline level (before the injection of carbamoyl-PROXYL). **(A)** Kinetic curves of normalized MRI signal intensity in kidneys before and after the injection of Gd-DTPA in ND mice **(A1)** or CD mice **(A2)**. The data are the mean from four animals (standard error did not exceed 20%). ImageJ software was used for data processing. **(B)** Typical kinetic curves of Gd-enhanced MRI in the ROI within the kidney of healthy humans **(B1)** and humans with renal pathology **(B2)**. CHOP-fMRU software was used for data processing of the postcontrast T_1_ VIBE Dynamic sequence in the coronal plane during excretory MR urography. CD, cholesterol diet; ND, normal diet [according to Tomizawa *et al.* (173)]. Color images are available online.

**FIG. 19.**
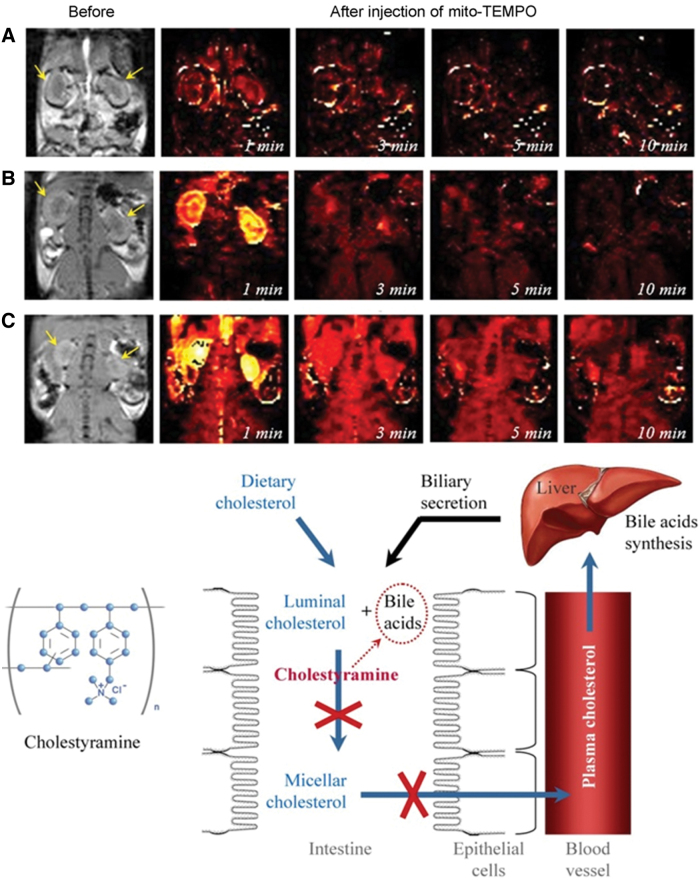
**Nitroxyl-enhanced MRI of the kidney in mice on a ND or high CD using mito-TEMPO as a contrast probe: black and white images: T_1_-weighted MR images of the kidney before the injection of mito-TEMPO.** Color images: extracted MRI signal intensity normalized to the averaged baseline level (before the injection of mito-TEMPO): **(A)** ND; **(B)** cholesterol plus cholestyramine diet; **(C)** CD. The *yellow arrows* indicate the kidneys. Structural formula and mechanism of reduced plasma cholesterol by the bile acid sequestrant cholestyramine [according to Lazarova *et al.* (103)]. Color images are available online.

One of the well-described models of renal dysfunction based on chronic inflammation is hypercholesterolemia-induced microvascularization in the renal cortex, and subsequent calcification that causes glomerulosclerosis and degeneration of the proximal tubules (92, 116, 152). In this experimental model, we did not detect nitroxyl-enhanced MRI signal in the kidneys of mice with hypercholesterolemia using carbamoyl-PROXYL as a contrast probe (Fig. 18) (173). However, a large decrease in renal perfusion was found using gadolinium-enhanced MRI. This suggests that the lack of nitroxyl-enhanced MRI signal of carbamoyl-PROXYL in the dysfunctional kidneys was not a result of rapid reduction of this nitroxyl radical to its diamagnetic form. In healthy mice, cell penetration of carbamoyl-PROXYL is limited *in vivo* due to the competition of this process with its relatively rapid excretion (79, 171). In contrast, using a hydrophobic and cell-penetrating nitroxyl radical, mito-TEMPO, we observed a long-lived nitroxyl-enhanced MRI signal in the kidneys of mice with hypercholesterolemia and short-lived MRI signal in the kidneys of healthy mice (Fig. 19) (103).

In this case, the probe was retained in the renal tissues, and the higher intensity of MRI signal of mito-TEMPO in the kidneys of mice with hypercholesterolemia was mainly due to the lower reducing capacity of inflamed renal tissues than the kidneys of healthy mice. This approach enables also the assessment of the effectiveness of antilipidemic drugs (Fig. 19).

The studies described above demonstrate the potential of nitroxyl probes for translational studies on functional MR urography (35, 38, 145). The major problem of this clinical approach is the use of contrast substances that increase the risk of intoxication in patients with impaired renal filtration. The efforts of clinicians are focused in two directions: (i) development of noncontrast methods for visualization and assessment of renal dysfunction; and (ii) development of nontoxic or low-toxicity contrast substances for functional urography (5, 8, 128). Low-toxicity nitroxyl-based contrast probes have the potential to meet the second condition. The structures of several TEMPO analog nitroxyl probes commonly used are shown in Supplementary Figure S2.

### Trials of redox imaging in the skin and other organs

The skin is the perfect target for EPRI of oxidative stress due to necessity of low depth of microwave penetration, which enables the use of S-band (2–3 GHz) for *in vivo* imaging or even X-band for isolated samples. After topical application of nitroxyl, its distribution in the skin and the redox status of the different layers can be visualized using a simple EPR spectral-spatial two-dimensional image. The potential of this approach has been well demonstrated in studies of human skin *in vivo* (95, 109, 154, 181), revealing opportunities for the investigation and diagnosis of skin pathologies, aging, and photo-induced damage.

Fuchs *et al.* (45) investigated the diffusion of several nitroxyl radical compounds into the skin of mice, and reported that lipophilic nitroxyl-labeled estradiol penetrates the dermis faster than hydrophilic nitroxyl-labeled procaine. The same group used X-band EPR to analyze the stability of nitroxyl compounds in skin biopsies, homogenates, and keratinocytes, and in the induction of erythema (43, 44). The rate of attenuation of the EPR signal of nitroxyl compounds increases in the following order: imidazoline < pyrrolidine < piperidine < oxazolidine (43). Thus, the nitroxyl compounds used either do not cause erythema, or the irritation is defined as moderate even at high concentrations (100 m*M*) (44). In this regard, one of the most popular nitroxyl radicals, TEMPOL, is used in ongoing clinical trials as a radioprotector for topical application, which prevents hair loss during radiation therapy of patients with malignancies (126).

Ultraviolet-induced overproduction of ROS in the skin has been also demonstrated using spin traps (85, 86) or nitroxyl probes (69–72, 167, 168, 170).

Another model of oxidative stress *in vivo* is the intoxication of animals with iron *via* the induction of Fenton reactions. Phumala *et al.* (144) conducted an EPR study on iron-loaded mice, and found that the EPR signal of carbamoyl-PROXYL in the liver decreased significantly faster than that in healthy animals, but this was suppressed by the pretreatment of mice with desferrioxamine or trolox. Similar dynamics of the EPR signal of carbamoyl-PROXYL were observed in the liver of animals exposed to ionizing radiation (131).

Han *et al.* (68) and Ahsan *et al.* (1) found that attenuation of the EPR signal of the nitroxyl radical CAT1 (4-trimethylammonium-2,2,6,6-tetramethylpiperidine-*N*-oxyl or choline-TEMPO) was significantly accelerated in the lungs of mice after intratracheal administration of burned diesel fuel particles. The effects of diesel particles were suppressed by scavengers of hydroxyl radicals and thiol-containing proteins (1, 68). It should be noted that CAT1 does not penetrate through cell membranes and cannot be an indicator of the intracellular redox status of alveolocytes. The dynamics of the EPR signal of CAT1 are most likely affected by extracellular substances such as ROS generated by the “oxidative burst” of macrophages in the pulmonary loci with concentrated diesel particles.

Leonard *et al.* (104) observed a significant delay in the reduction of the nitroxyl probe TEMPOL in the lungs of asbestos-treated mice. Caia *et al.* (15) reported that mice exposed to cigarette smoke had lower reducing capacity toward the carbamoyl-PROXYL in almost all abdominal organs.

Togashi *et al.* (172) also used the carbamoyl-PROXYL and EPRI system to analyze the redox status of the liver in carbon tetrachloride (CCl_4_)-treated mice. They reported that the rate of nitroxyl reduction was significantly slower in CCl_4_-treated mice than in untreated controls. Another EPR study of mice with hepatic injury (as a result of ischemia–reperfusion) reported that blockade of Ca^2+^/calmodulin by the calmodulin antagonist CV159 resulted in faster attenuation of the EPR signal of carbamoyl-PROXYL in treated animals than in untreated controls (96).

### Possibilities of redox imaging of biological objects using spin trapping

EPR spin-trapping techniques are attractive modalities, as they enable the detection of specific ROS. The *in vivo* detection of free radicals by spin trapping was previously demonstrated in mice irradiated with ionizing radiation, which induces the overproduction of ROS and severe oxidative stress (66, 67). Visualization of O_2_^•−^ and hydroxyl radicals can be improved by developing more stable spin traps and the use of ^15^N-substituted spin trapping, which may increase the sensitivity and resolution by reducing the number of lines in the EPR spectrum (94). However, imaging biological ROS using a combination of *in vivo* spin trapping and EPRI and/or MRI is difficult at this time.

Over the last decade, an immunospin capture method was developed based on the concept that DMPO (5,5-dimethyl-1-pyrroline-*N*-oxide) reacts with protein radicals, and the products can be identified immunologically with high specificity. Mason (115) developed antibodies against DMPO–protein radical adducts (anti-DMPO) that can be used in immunoblotting, immunohistochemistry, immunofluorescence, and flow cytometric analyses. This approach significantly extends the benefits of using spin traps in the detection of redox-active substances because immunological techniques are characterized by high sensitivity (17, 115, 146). Immunospin traps have been used to detect DMPO–protein products of myoglobin and hemoglobin, as well as ROS adducts in isolated mitochondria, cells, and tissue samples (17, 26, 59, 60, 100).

However, the method is only applicable *in vitro* and has limitations: (i) it cannot detect free radicals, such as O_2_^•−^ and hydroxyl radical, and only enables the identification of modified protein adducts; (ii) multiple antibodies specific for different types of protein adducts are required to cover the full range of targets to ensure high sensitivity of the analysis.

### Redox imaging of biological objects using cyclic hydroxylamines

**FIG. 20.**
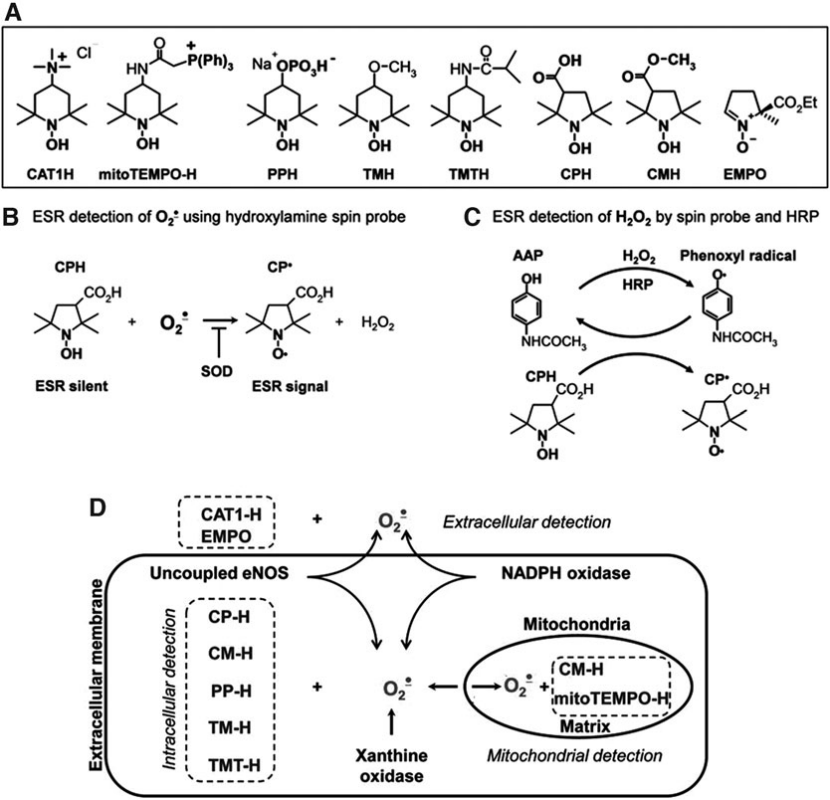
**Detection of mitochondrial and cellular ROS by hydroxylamine probes. (A)** Chemical structures of hydroxylamine probes. **(B)** EPR detection of cellular superoxide using hydroxylamines. **(C)** EPR detection of hydrogen peroxide using hydroxylamines and HRP. **(D)** General scheme of EPR detection of superoxide in extracellular, intracellular, or mitochondrial compartments using cyclic hydroxylamine spin probes. HRP, horseradish peroxidase; Rot, rotenone. [Adapted from Dikalov *et al.* (32) and Dikalov and Harrison (31).]

At the end of the last century, diamagnetic forms of nitroxyl radicals, cyclic hydroxylamines (Fig. 20), were found to be suitable for the detection of O_2_^•−^ in biological objects *in vitro* and *in vivo* (27, 29–33). Hydroxylamines are diamagnetic and have no EPR/MRI contrast. They are oxidized by ROS (in particular, by O_2_^•−^) with the formation of stable paramagnetic substances, nitroxyl radicals, with a half-life of up to several hours in isolated model and biological systems (27, 29–31). This enables them to be registered by EPR spectroscopy, EPRI, and MRI in the case of oxidation.

Compared with nitrons, cyclic hydroxylamines react much faster with O_2_^•−^ radicals, with a reaction rate constant of ∼10^3^–10^4^ *M*^−1^·s^−1^ at pH = 7.4. This favors the competition of hydroxylamines with cellular antioxidants and improves the efficiency of detection of intracellular O_2_^•−^ (30). For this reason, hydroxylamines can be used at relatively low concentrations (0.05–1 m*M*), which minimizes their side effects in biological systems. Another advantage of cyclic hydroxylamines is that they react with O_2_^•−^ in a single chemical reaction. This reduces the potential artifacts arising from multistage redox reactions typical of other redox-sensitive probes (fluorescent and chemiluminescent) (42). For example, unlike lucigenin, cyclic hydroxylamines do not have the so-called “redox cycling,” and no additional ROS were generated (114).

Dikalov *et al.* (32) investigated the dynamics of the EPR signal of cyclic hydroxylamines in biological systems. This study supports the essential role of the hydrophobic (amphiphilic) nature of cyclic nitroxyl probes, and their penetration into cells in the dynamics of their contrast properties and their ability to be used as intracellular or extracellular redox sensors. The authors demonstrated that cationic and membrane-impermeable CAT1-H (hydroxylamine form of CAT1) detects only extracellular O_2_^•−^ released from live cells or extramitochondrial O_2_^•−^ released from isolated mitochondria. In contrast, cell-penetrating MCP-H (hydroxylamine form of MC-PROXYL), PP-H (hydroxylamine form of 4-phosphonooxy-TEMPO), and mito-TEMPO-H (hydroxylamine form of mito-TEMPO) are suitable for the detection of intracellular and extracellular ROS. PP-H is thought to accumulate in cells by active transport, MCP-H accumulates predominantly in the cytoplasm, and mito-TEMPO-H is mitochondrially targeted and localized predominantly in mitochondria (32, 84). MCP-H may also accumulate in mitochondria.

MCP-H gives a strong EPR signal in cells treated with phorbol-12-myristate-13-acetate, stimulating the production of cytoplasmic O_2_^•−^, whereas the signal of mito-TEMPO-H is weaker in this case (25, 32). In contrast, rotenone-induced generation of mitochondrial O_2_^•−^ results in a strong EPR signal induced by mito-TEMPO-H and MCP-H, but not PP-H (32). Thus, relatively accurate information on the origin of ROS (extracellular, intracellular, or mitochondrial) can be obtained using appropriate selection of cyclic hydroxylamine.

In general, the advantage of cyclic hydroxylamines is in their use for direct detection of ROS in cells, subcellular fractions, and tissue homogenates at room temperature by examining the accumulation of the nitroxyl radical by EPR spectroscopy. Low-temperature EPR is suitable for detecting ROS in intact tissues. For this purpose, the samples were incubated in physiological saline containing cyclic hydroxylamines and then frozen in liquid nitrogen, stored at −80°C, and later analyzed at a low temperature (9, 28).

Kozlov *et al.* (98) injected cyclic hydroxylamines into young and old rats, and monitored the appearance and increase in EPR signal in tissue homogenates isolated from various organs *ex vivo*. The authors found that the oxidative capacity of the blood, skeletal muscle, lungs, and heart increases significantly with age, but does not change the redox status of the intestine, brain, liver, or kidneys. Cyclic hydroxylamines have been used in model systems and isolated biological objects, including blood and biopsy samples from patients with numerous diseases (9, 113). However, no use of these spin probes in nitroxyl-enhanced EPRI or MRI has been reported in intact animals *in vivo*.

### Other contrast substances and techniques for redox imaging and perspectives on cyclic nitroxyl radicals

There are many contrast media that can give detectable products reflecting the localization and level of a particular target investigated; that is, the redox-active molecular species or the biological redox environments. The most of *in vitro* detection of these contrasts (*e.g*., fluorescence) is feasible with high sensitivity and resolution, but *in vivo* detection is highly difficult to implement. Another group of contrast agents (*e.g*., nuclear and ultrasound) can achieve *in vivo* detection with high sensitivity, although the resolution is low. In general, nuclear-labeled contrast agents can provide indirect information about the tissue redox status, which can be given as a result of biochemical and physiological processes such as glycometabolism, O_2_ consumption, hypoxia, and cell retention depending on the cytoplasmic redox potential. In addition, such radioactive contrast agents may increase the risks for the patient. Of note, the above-mentioned methodologies enable assessment of the redox status of the biological object based on the information obtained for one or several redox-active compounds. Thus, the discussions and conclusions in studies are often contradictory.

At present, efforts are focused on mapping the redox status of tissues and organs in intact organisms. The perfect methodology should provide direct and noninvasive detection of the redox status of the target organ *in vivo*. In this context, the perfect redox-sensitive contrast substances should meet the following conditions:

- Ability to penetrate the cells and BBB, if possible;
- ability to provide information about the equilibrium between the intracellular oxidizers and reducers for the total redox status of cells and tissues, not only for the status of a certain redox-active compound (*e.g*., its oxidized or reduced form);
- be nontoxic or low-toxic *in vivo*;
- be rapidly excreted through the living organism;
- have high contrast and enable imaging with high resolution.

Cyclic nitroxyl radicals have a relatively low toxicity (safer than gadolinium and manganese complexes) and are not mutagenic (2, 21). They are characterized by favorable biomedical effects such as anticancer effects, regulation of body weight, protection against ischemia–reperfusion injury, protective effects against cataract, sensitizing cancer cells and tissues to ionizing radiation, and protecting normal cells and tissues (34, 107, 163, 164, 200). Moreover, some cyclic nitroxyl radicals are undergoing clinical trials for topical applications (192). This confirms the potential of nitroxyl radicals as new contrast substances for redox imaging in translational studies on humans using MRI. However, this can only be achieved after many preliminary studies on experimental animals to select the most appropriate nitroxyl probes for redox imaging, route of administration, and safe doses. This will provide a new opportunity for MRI/EPRI analysis of metabolic pathways, accompanied by minor changes in the redox status of biological objects and induction of oxidative stress.

## Conclusion

Redox imaging is a useful tool to detect an abnormal tissue redox status such as disordered oxidative stress or tumor hypoxia. Data from nitroxyl-enhanced MRI/EPRI *in vivo* must be considered and interpreted carefully because the kinetics of the signal in the target tissue or organ depend on a number of factors: (i) life time of nitroxyl radicals in the bloodstream; (ii) penetration through cell membranes and localization in target cells and tissues; (iii) rate of excretion from the organism; (iv) selection of appropriate ROI; and (v) use of healthy individuals as controls. A suitable chemical and/or biological nitroxyl radical contrast agent will provide useful information for translational theranostic applications in the target organ/tissue.

## Supplementary Material

Supplemental data

Supplemental data

## Abbreviations Used

^•^H: hydrogen radicals
^•^OH: hydroxyl radicals
23c: 4-(*N*-methylpiperidine)-2,2,5,5-tetramethylpyrroline-*N*-oxyl
2D: two dimensional
3D: three dimensional
AA: ascorbic acid
BBB: blood–brain barrier
carbamoyl-PROXYL: 3-carbamoyl-2,2,5,5-tetramethylpyrrolidine-*N*-oxyl
carboxy-PROXYL: 3-carboxy-2,2,5,5-tetramethylpyrrolidine-*N*-oxyl
CAT1: 4-trimethylammonium-2,2,6,6-tetramethylpiperidine-*N*-oxyl
CCl_4_: carbon tetrachloride
CD: cholesterol diet
CW: continuous wave
CxP-Am: acetoxymethyl-2,2,5,5-tetramethylpyrrolidine-*N*-oxyl-3-carboxylate
DMPO: 5,5-dimethyl-1-pyrroline-*N*-oxide
DNP: dynamic nuclear polarization
DT: dopamine transporter
EPR: electron paramagnetic resonance
EPRI: EPR imaging
FA: flip angle
FLASH: fast low angle shot
H_2_O_2_: hydrogen peroxide
HM-PROXYL: 3-(hydroxymethyl)-1-oxy-2,2,5,5-tetramethylpyrrolidine
HO_2_^•^: hydroperoxyl radical
HRP: horseradish peroxidase
KA: kainic acid
KIE: kinetic isotope effects
LPS: lipopolysaccharides
MCP-H: hydroxylamine form of MC-PROXYL
MC-PROXYL: 3-methoxycarbonyl-2,2,5,5-tetramethylpyrrolidine-*N*-oxyl
mito-TEMPO: 2,2,6,6-tetramethyl-4[[2-(triphenylphosphonio)acetyl]amino]-1-piperidinyloxy
mito-TEMPO-H: hydroxylamine form of mito-TEMPO
MPP^+^: 1-methyl-4-phenylpyridinium
MPTP: 1-methyl-4-phenyl-1,2,3,6-tetrahydropyridine
MR: magnetic resonance
MRI: magnetic resonance imaging
ND: normal diet
O_2_: oxygen
O_2_^•−^: superoxide
OMRI: Overhauser-enhanced MRI
pO_2_: partial oxygen pressure
PP-H: hydroxylamine form of 4-phosphonooxy-TEMPO
ROI: region of interest
ROO^•^: peroxyl radical
ROS: reactive oxygen species
Rot: rotenone
RS^•^: thiyl radicals
SD: standard deviation
SG: salivary gland
SLENU: TEMPO-labeled nitrosourea
*SNpc*: *substantia nigra pars compacta*
TE: echo time
TEMPO: 2,2,6,6-tetramethylpiperidine-*N*-oxyl
TEMPO^+^: oxoammonium form of TEMPO
TEMPO-H: hydroxylamine form of TEMPO
TEMPOL: 4-hydroxy-2,2,6,6-tetramethylpiperidine-*N*-oxyl
TES: 4-[(3-carboxy-1-oxopropyl)amino]-2,2,6,6-tetramethyl-1-piperidinyloxy
TR: repetition time
