# Supplementary material for: Nitroxyl Radical as a Theranostic Contrast Agent in Magnetic Resonance Redox Imaging
Source: Antioxid Redox Signal. 2022 Jan 17;36(1-3):95–121. doi: 10.1089/ars.2021.0110 (PMC8792502; doi:10.1089/ars.2021.0110)
Supplement: Supplemental data [file Supp_FigS1.docx]

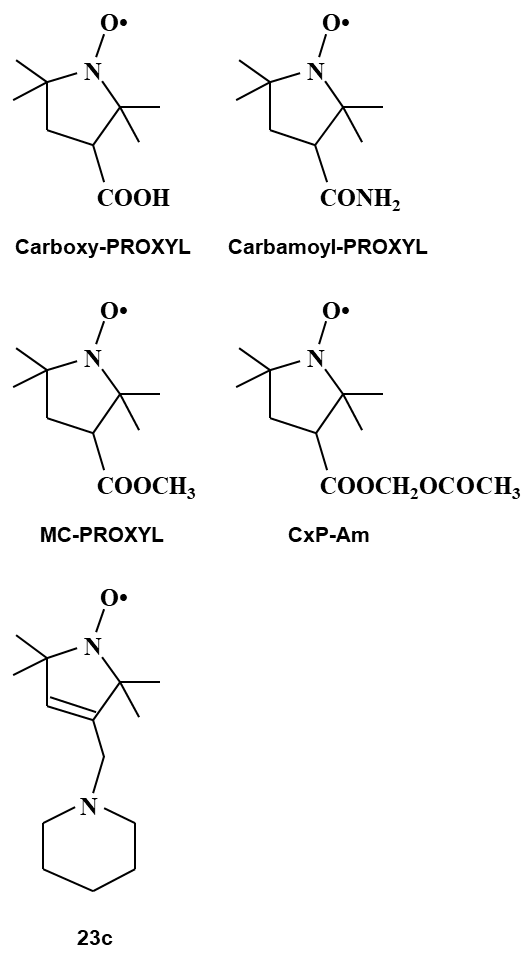


Suppl. Fig. 1. Structures of 5-memberd ring (pyrrolidine or pyrroline) nitroxyl contrast agents introduced in this review. The pyrrolidine-type nitroxyl radicals are called PROXYL.
