# Supplementary material for: Nitroxyl Radical as a Theranostic Contrast Agent in Magnetic Resonance Redox Imaging
Source: Antioxid Redox Signal. 2022 Jan 17;36(1-3):95–121. doi: 10.1089/ars.2021.0110 (PMC8792502; doi:10.1089/ars.2021.0110)
Supplement: Supplemental data [file Supp_FigS2.docx]

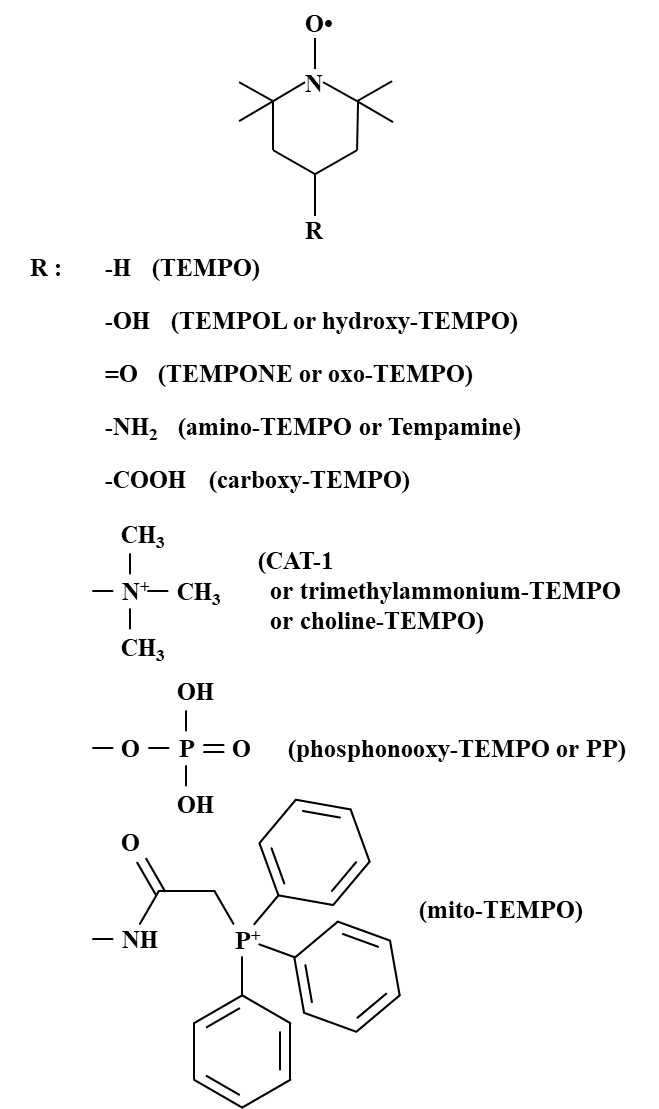


Suppl. Fig. 2. Structure of 6-memberd ring (TEMPO) nitroxyl radicals and the analogues introduced in this review.
